# Identification of a robust promoter in mouse and human hepatocytes by *in vivo* biopanning of a barcoded AAV library

**DOI:** 10.1016/j.ymthe.2025.04.027

**Published:** 2025-04-21

**Authors:** Jonas Becker, Claire Domenger, Pervinder Choksi, Chiara Krämer, Conradin Baumgartl, Olena Maiakovska, Jae-Jun Kim, Jonas Weinmann, Georg Huber, Florian Schmidt, Christian Thirion, Oliver J. Müller, Holger Willenbring, Dirk Grimm

**Affiliations:** 1 Department of Infectious Diseases/Virology, Section Viral Vector Technologies, Medical Faculty, University of Heidelberg, 69120 Heidelberg, Germany; 2 BioQuant, Center for Integrative Infectious Diseases (CIID), University of Heidelberg, 69120 Heidelberg, Germany; 3 Department of Surgery, Eli and Edythe Broad Center of Regeneration Medicine and Stem Cell Research, University of California, San Francisco (UCSF), San Francisco, CA, USA; 4 Revvity Gene Delivery GmbH, 82166 Gräfelfing, Germany; 5 Department of Internal Medicine V, University Hospital Schleswig-Holstein and University of Kiel, 24105 Kiel, Germany; 6 German Centre for Cardiovascular Research (DZHK), partner site Hamburg/Kiel/Lübeck, 24105 Kiel, Germany; 7 German Center for Infection Research (DZIF) and German Center for Cardiovascular Research (DZHK), partner site Heidelberg, 69120 Heidelberg, Germany; 8 Faculty of Engineering Sciences, University of Heidelberg, 69120 Heidelberg, Germany; 9 These authors contributed equally; 10 Present address: Novartis Pharma, Fabrikstrasse 2, 4056 Basel, Switzerland; 11 Present address: Department of Biosystems Science and Engineering, ETH Zurich, Klingenbergstrasse 48, 4056 Basel, Switzerland

## Abstract

Recombinant adeno-associated viruses (AAVs) are leading vectors for *in vivo* human gene therapy. An integral vector element is promoters, which control transgene expression in either a ubiquitous or cell-type-selective manner. Identifying optimal capsid-promoter combinations is challenging, especially when considering on- versus off-target expression. Here, we report a pipeline for *in vivo* promoter biopanning in AAV building on our AAV capsid barcoding technology and illustrate its potential by screening 53 promoters in 16 murine tissues using an AAV9 vector. Surprisingly, the 2.2-kb human glial fibrillary acidic protein (GFAP) promoter was the top hit in the liver, where it outperformed robust benchmarks such as the human α−1-antitrypsin promoter or the clinically used liver-specific promoter 1 (LP1). Analysis of hepatic cell populations revealed preferred GFAP promoter activity in hepatocytes. Notably, the GFAP promoter also surpassed the LP1 and cytomegalovirus promoters in human hepatocytes engrafted in an immune-deficient mouse. These findings establish the GFAP promoter as an exciting alternative for research and clinical applications requiring efficient and specific transgene expression in hepatocytes. Our pipeline expands the arsenal of technologies for high-throughput *in vivo* screening of viral vector components and is compatible with capsid barcoding, facilitating the combinatorial interrogation of complex AAV libraries.

## INTRODUCTION

Adeno-associated virus (AAV) continues to excel as a vector for local or systemic gene therapy in humans, exemplified by the recent market approval of the intravenously applied AAV-based drugs Elevidys and Beqvez.^1,2^ However, despite a plethora of clinical data supporting the overall safety, efficacy, and tolerability of AAV vectors, there are increasing reports of toxicity or even fatalities after high-dose administration of AAV vectors in large animals and humans.^3–5^ These alarming events demonstrate the urgent need for safer, more efficient, and more targeted vectors that achieve therapeutic levels of (persistent) transgene expression at lower doses.

To identify these next-generation AAVs, many laboratories including ours explore naturally occurring capsid variants and harness their amenability to genetic engineering or chemical repurposing.^6–9^ As illustrated by a wealth of studies, genuine or synthetic AAV capsids can enable targeted and/or robust transgene delivery to selected organs and cells across species, from local or systemic delivery. Capsid performance can be enhanced further by combining transductional targeting with vector fine-tuning on the transcriptional level by employing tissue- or cell-type-specific promoters and/or enhancers.^10–14^ Additional control at the mRNA level can be achieved through splicing via synthetic introns or enhancing stability using different polyadenylation sites or the woodchuck hepatitis virus post-transcriptional regulatory element.^11,15,16^ Cell targeting can also be optimized at the post-transcriptional level by tagging an expression cassette with binding sites for microRNAs (miRNAs) that are abundant in off-target cells, where they will silence the vector-expressed mRNA in the case of inadvertent transduction.^17–19^

In particular, transcriptional targeting has flourished over the last several years, concurrent with our expanding toolbox of *cis*-acting elements that were discovered experimentally or bioinformatically, individually, or in high-throughput screens.^10,20^ Before this, AAV vectors typically employed strong and ubiquitously active promoters such as the immediate-early enhancer and promoter combination from cytomegalovirus (CMV) or a CMV enhancer fused to the promoter and first intron of chicken β-actin (CAG),^21,22^ which achieve high levels of transgene expression irrespective of the transduced cell type or tissue but are subpar in clinical applications where vector specificity and, related to this, safety are prime concerns. This is especially critical when combining systemically applied and promiscuous capsids, such as AAV9, with cargoes that are prone to exacerbating safety concerns if not controlled properly, such as CRISPR or RNAi.^23,24^ The ability to achieve organ- or even cell-type-specific AAV cargo expression is also crucial for transgene products that are secreted from the transduced cells, such as the blood coagulation factors VIII or IX, as their post-translational modification depends on their cellular origin. This is why, for instance, the AAV5-based therapeutic Hemgenix for hemophilia B treatment uses the liver-specific promoter 1 (LP1)—consisting of the human apolipoprotein E/C-I gene locus, the human α−1-antitrypsin (AAT) promoter, and the minute virus of mice intron—to limit factor IX expression to hepatocytes as its natural production site.^25^

However, finding the optimal capsid-promoter combination for a given application is far from trivial for several reasons. The design of synthetic promoters (i.e., identifying and combining *cis*-regulatory elements [CREs] with core promoters) is challenging and not well understood, thus requiring intensive follow-up testing of promoter activity.^26^ Furthermore, there is an overwhelming wealth of available promoters of human, viral, or synthetic origin, whose systematic individual testing in combination with specific capsids would entail excessive numbers of vector preparations and, when tested *in vivo*, extreme numbers of animals. The latter is exemplified by a study in which 230 AAV vectors encoding distinct promoter elements were injected individually into a total of 920 mouse eyes (4 eyes per vector), exemplifying the excessive number of animals needed for such experiments.^12^ Moreover, akin to AAV capsid performance, promoter activities can vary substantially between cells in culture and cells or whole organs *in vivo*, increasing the need for creative high-throughput solutions that overcome all these issues with individual testing schemes in animals.^27–29^

As a major step in this direction, several groups devised and harnessed massively parallel reporter assays (MPRAs) that enable the concurrent and unbiased screening of millions of putative CREs in cells or animals.^30–32^ These MPRA libraries are usually either barcoded or self-transcribing. Barcoded libraries link each CRE to a specific barcode in the untranslated region (UTR) of a reporter to later assess transcriptional activity by next-generation sequencing (NGS),^30–32^ while self-transcribing libraries include the CRE as part of the UTR.^33,34^ MPRAs are then executed by the transfection of cells *in vitro* or by *in vivo* delivery to mice as naked DNA through hydrodynamic tail vein injection or encapsidated in AAV. Following delivery, DNA and RNA are extracted from the transfected/transduced cells and subjected to high-throughput amplicon sequencing to comparatively assess the contribution of different CRE variants to RNA output. Recently, others created focused barcoded AAV libraries comprising a small set of previously characterized or rationally engineered promoters and then screened them in selected organs in mice or non-human primates.^35,36^

Here, we report a comprehensive barcoded library of 53 distinct promoter variants that are commonly employed in AAV vectors and illustrate the results of its screening in an AAV9 context in 16 major tissues following systemic delivery in adult mice. To this end, we repurposed our barcoding strategy previously used for capsid screening, where a barcode in the 3′ UTR of an AAV reporter cassette enables the qualitative and quantitative assessment of the activity of its associated capsid or now promoter, respectively, by NGS-based barcode readout on the DNA (i.e., vector genome [VG] delivery) and RNA (i.e., functional activity resulting in transcription) level amid a pool of variants produced and injected simultaneously.^37^ In the present study, this allowed us to identify the previously unknown high and specific *in vivo* activity of the 2.2-kb human glial fibrillary acidic protein (GFAP) promoter^38^ in the mouse liver and, more specifically, in hepatocytes not only of mouse but also of human origin. This finding showcases the potential of our pipeline and argues for further developing and using the GFAP promoter for AAV-based *in vivo* liver gene therapies requiring high hepatocyte specificity.

## RESULTS

### *In vivo* biopanning of an AAV promoter library in adult mice

**Figure 1.**
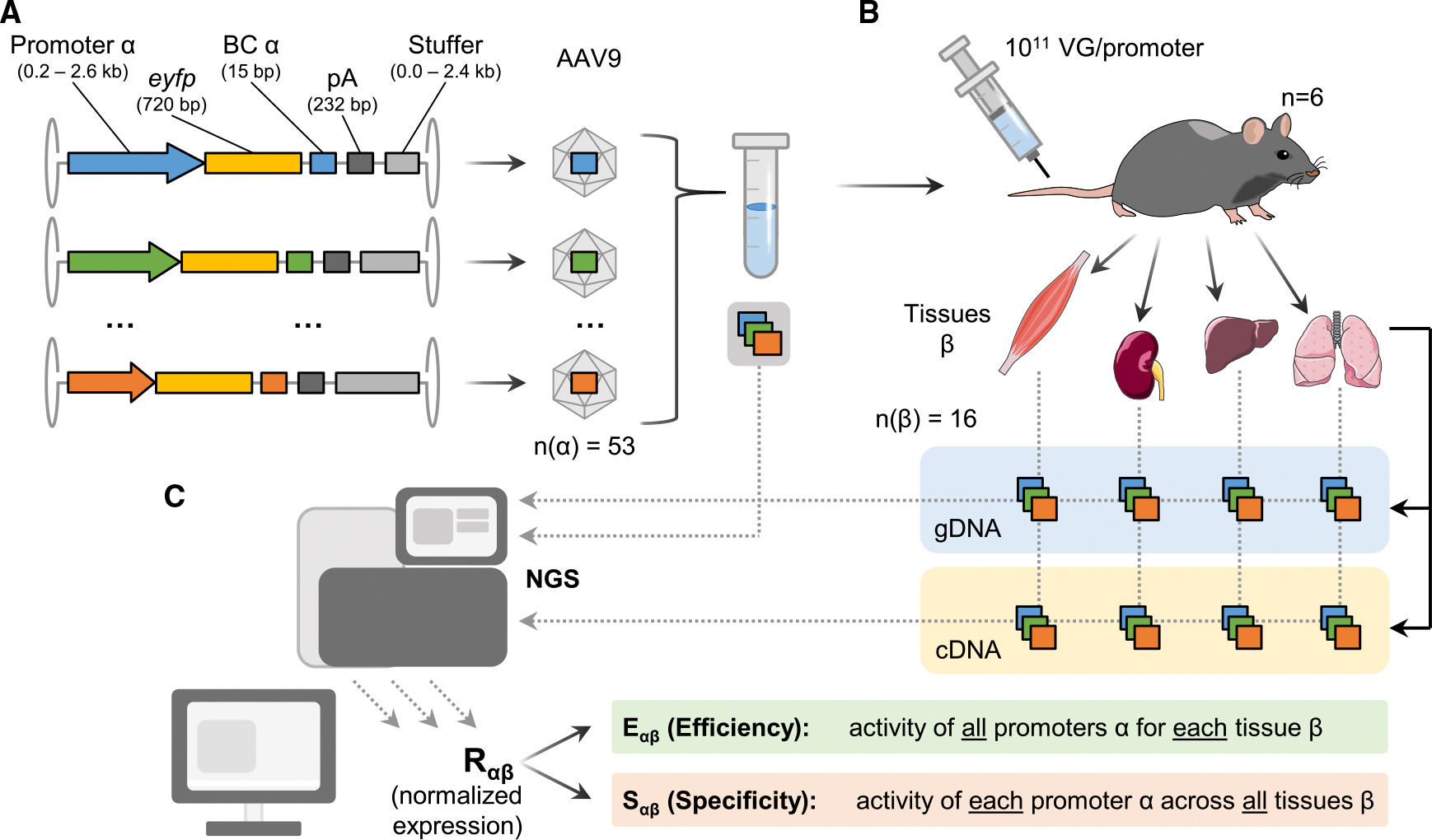
Barcode-based parallel evaluation of the *in vivo* expression efficiency and specificity of a library of 53 promoters (A) Single-stranded AAV vectors were engineered to contain a promoter of choice and an associated barcode (BC) in the 3′ untranslated region of an *eyfp* transgene to allow monitoring of both biodistribution and expression. Different stuffer sequences were included based on promoter size to ensure comparable overall construct sizes. All 53 barcoded promoter constructs were individually packaged in AAV9 capsids, titrated, and mixed in equal amounts. pA, polyadenylation signal. (B) The resulting promoter library was systemically injected into female C57BL/6 mice (*n* = 6) at a dose of 10^11^ VG per promoter and per mouse. Two weeks post-administration, the mice were sacrificed, and 16 different tissues were isolated. From each tissue, genomic (g)DNA and RNA were extracted, followed by reverse transcription of RNA to complementary (c) DNA. (C) Illumina sequencing was used to assess barcode frequencies within the starting promoter library, as well as within the gDNA and cDNA samples. The resulting BC counts were used to calculate the normalized expression of each promoter *α* within each tissue *β* (see materials and methods and Figure S1 for details). This normalized expression was then deconstructed into efficiency and specificity scores by normalizing across all promoters for each tissue or by normalizing across all tissues for each promoter, respectively.

We created an AAV promoter library using 53 different RNA polymerase II promoters or enhancer-promoter combinations that have previously been reported to possess distinct organ- or cell-type-specificities or ubiquitous expression profiles (detailed in Table S1). All sequences were cloned into a single-stranded AAV backbone in front of a reporter cassette comprising an enhanced yellow fluorescent protein (*eyfp*) transgene, a unique barcode of 15 nucleotides assigned to each promoter in the 3′ UTR, and a robust bovine growth hormone (bGH) polyadenylation signal (Figure 1A). Finally, we added various stuffers derived from the *lacZ* coding sequence (Table S1) to ensure similar overall insert sizes. All 53 resulting AAV VGs containing unique promoter-barcode combinations were separately packaged in AAV9. This serotype was chosen for its broad tropism in mice,^37,39^ enabling comprehensive *in vivo* evaluation of promoter performance in a wide variety of tissues and cells, including those in which the selected promoters were expected to be active. After individual vector purification and titration, equal amounts of VGs per construct were pooled. Homogeneous pooling was confirmed by NGS of the barcode region of the packaged vectors within the resulting viral library (Figure S1).

This library was then systemically delivered to six female C57BL/6 mice by tail vein injection at a dose of 1 × 10^11^ VGs per promoter construct per mouse (corresponding to a total dose of 5.3 × 10^12^ VGs/mouse) (Figure 1B). Two weeks later, the animals were sacrificed and 16 major tissues—brain, diaphragm, eye, fat, gut (duodenum), heart, kidney, liver, lung, lymph nodes (inguinal), skeletal muscle (*quadriceps femoris*), ovary, pancreas, skin, spleen, and stomach—collected. NGS-based readout of the barcode region allowed tracing of promoter constructs on the genomic (g) DNA and mRNA (converted to complementary [c]DNA) levels using our in-house workflow previously reported in the context of barcoded AAV capsid libraries.^37,40–42^ The corresponding normalization strategy was adapted to the current data by normalizing cDNA reads (expression at the mRNA level) within each tissue to the biodistribution of each barcoded vector (amplified from gDNA) in the same tissue. Additional normalization by quantitative (qPCR) real-time PCR to (1) the bulk *eyfp* mRNA expression measured in the cDNA of each tissue and (2) a reference housekeeper (*PolR2A*) yielded a normalized expression value Rαβ. This allowed us to assess not only promoter efficiency within each organ (efficiency scores Eαβ) but also promoter specificity by comparing promoter expression levels across tissues (specificity scores Sαβ; Figure 1C).

**Figure 2.**
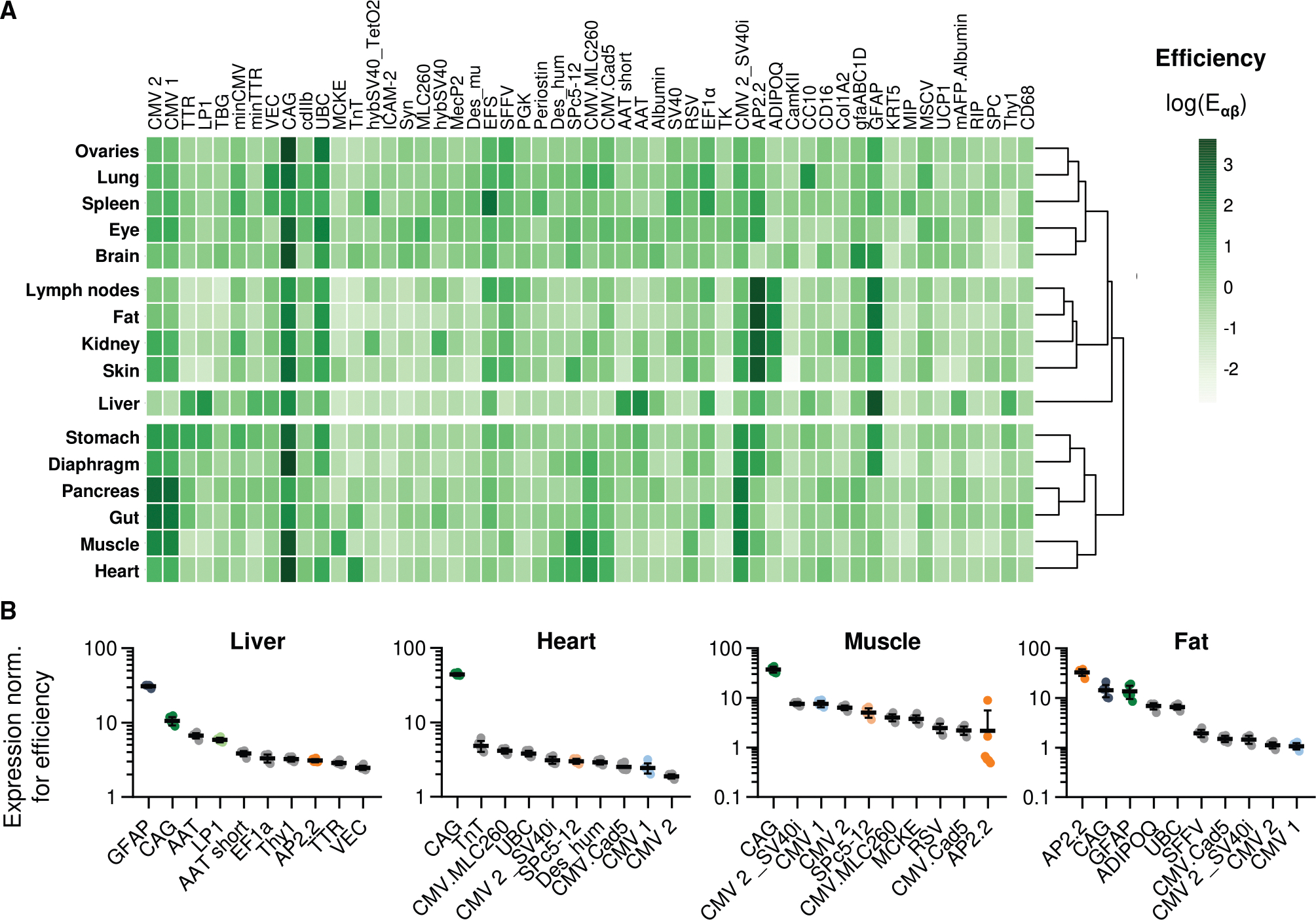
Efficiencies of 53 promoters in 16 tissues (A) Unsupervised hierarchical cluster heatmap of normalized expression levels deconstructed into efficiency scores. Shown are logarithmic efficiency scores averaged from six animals. (B) Efficiency values for the 10 strongest promoters in liver, heart, muscle, or fat tissue are shown as mean ± standard deviation. Complete efficiency scores of all promoters for each tissue are presented in Figures S2–S5.

The established efficiency scores are useful to identify the strongest expressing promoters in each tissue and thus help to find the most appropriate expression cassette that drives maximum transcription in a chosen target tissue (Figure 2). For 8 of the 16 assessed organs, the 1.6-kb CAG promoter^21^ demonstrated the highest average efficiency scores (Figure 2A). It was only surpassed by the murine adiponectin AP2.2 promoter,^43,44^ which yielded higher expression in fat, kidney, lymph nodes, and skin tissue; by the CMV promoter^22,37^ variants CMV1, CMV2, and CMV2_SV40i in the pancreas and gut; by the short eukaryotic translation elongation factor 1 α1 promoter^45^ in the spleen; and by the GFAP promoter^38^ in liver tissue. Figure 2B visualizes the measured promoter efficiencies by showing a ranking of the top 10 promoter variants in 4 tissues that showed high overall expression levels (see also Figure 2A). A complete overview of the efficiency of all 53 promoters in all 16 tissues is found in Figures S2–S5.

**Figure 3.**
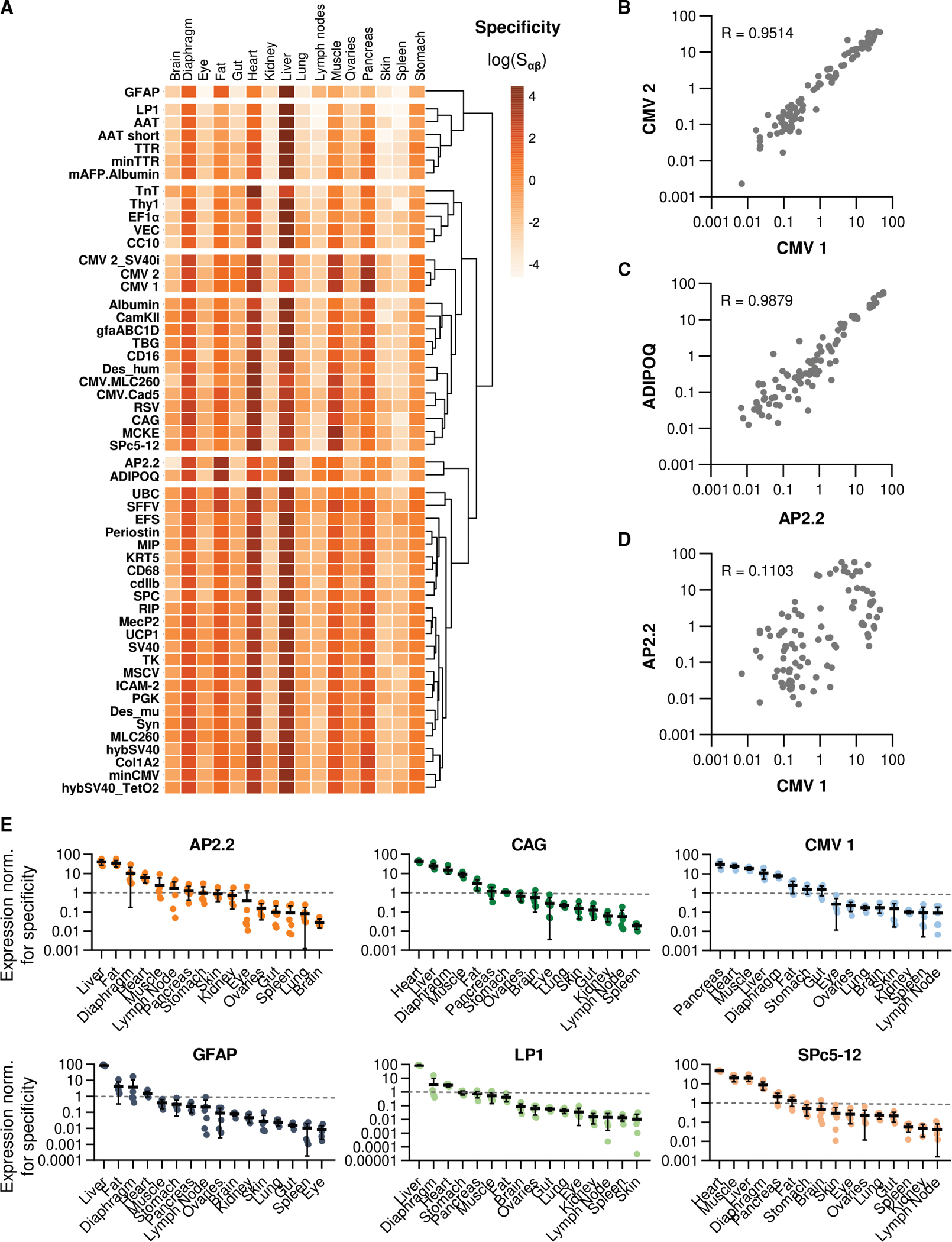
Specificity of each promoter across different tissues (A) Heatmap of mean specificity scores for each of the 53 promoters using unsupervised hierarchical clustering. (B–D) Correlation of specificity scores matched between promoters (B) CMV1 and CMV2, (C) AP2.2 and ADIPOQ, or (D) CMV1 and AP2.2. Each dot represents the specificity score of the two compared promoters for one tissue from one of six replicate animals. Pearson correlation coefficients R were calculated using GraphPad Prism. (E) Specificity of the promoters AP2.2, CAG, CMV1, GFAP, LP1, and SPc5–12 ranked by the strength of expression in the tissues shown. Values are expressed as mean ± standard deviation. Complete specificity scores for all promoters are presented in Figures S6–S8.

This analysis of promoter efficiency is complemented by the evaluation of the specificity of each promoter across the 16 tissues tested (Figure 3). By allowing for an assessment of potential off-target expression rates, these specificity scores revealed that no promoter variant was exclusively active in a single tissue. After delivery with AAV9, all tested promoters provided strong *eyfp* expression in the liver, which was the dominant source of expression for 42 out of the 53 tested promoter constructs (Figure 3A). Comparing specificity scores between different promoters (Figures 3B–3D) showed good correlations for related promoters, such as two versions of the CMV promoter (CMV1 and CMV2, Figure 3B) or the two fat-targeted adiponectin promoters AP2.2 (mouse)^43^ and ADIPOQ (human; Figure 3C).^46^ As expected, comparing specificities for two unrelated promoters, such as CMV1 and AP2.2, showed no such correlation (Figure 3D). Specificity scores for all tissues are shown for 6 selected promoters in Figure 3E and for all 53 in Figures S6–S8.

Collectively, our results confirm that widely used promoter variants such as CAG, ubiquitin C,^47^ or CMV are both robust and ubiquitously active. Furthermore, our data confirm the high efficiency and specificity of the LP1 promoter in the liver^25^ or the SPc5–12 promoter in different striated muscle tissues (heart, skeletal muscle, and diaphragm).^48^ It is noteworthy that although these promoters were less efficient than the CAG promoter in these tissues (Figure 2), they were more specific (Figure 3). Surprisingly, the 2.2-kb human GFAP promoter was most efficient at expressing in the liver, which was unexpected because it is commonly known for its astrocyte-specific expression in the central nervous system (CNS).^38,49,50^ In fact, in our library, GFAP outperformed not only CAG but also all liver-specific promoters in hepatic expression, including the AAT,^51^ LP1, and transthyretin^52^ promoters (Figures 2A and 2B).

### Validation of promoter library data via single-vector injections

**Figure 4.**
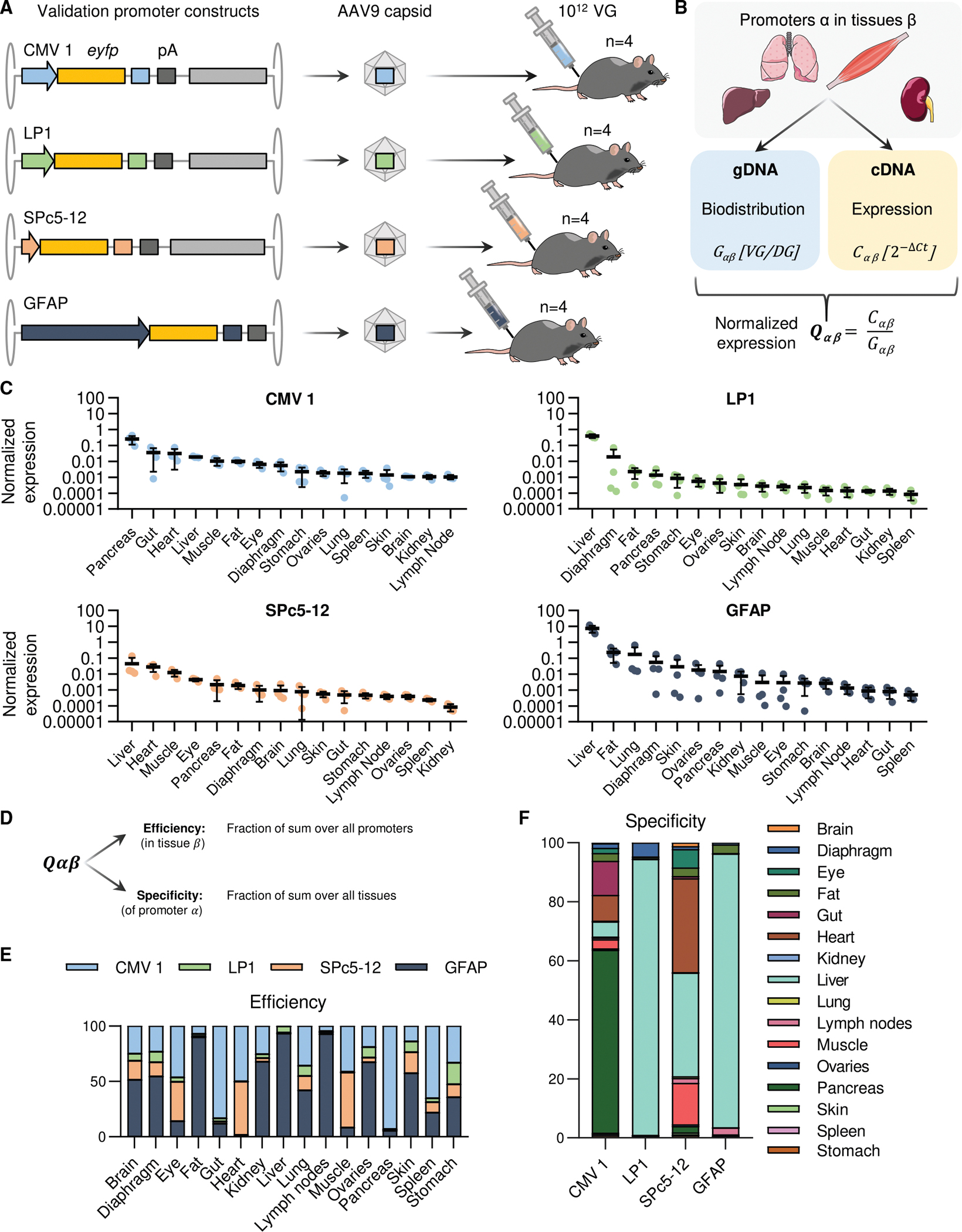
Individual validation of the activity of the CMV1, LP1, SPc5–12 and GFAP promoters (A) Individual promoter constructs were packaged into AAV9 and injected into female C57BL/6 mice (*n* = 4 mice per group) at a dose of 10^12^ VG/mouse. (B) Two weeks post-administration, tissues were harvested and subjected to DNA and RNA extraction; the latter was followed by cDNA synthesis. Biodistribution *Gαβ* of AAV vectors was measured by ddPCR from gDNA (viral genomes per diploid genome), and expression *Cαβ* was measured by qPCR from cDNA (2^−ΔCt^). Normalized expression *Qαβ* was calculated by dividing the expression driven by promoter *α* in each tissue *β* by the respective biodistribution values. (C) Normalized expression *Qαβ* for the CMV1, LP1, SPc5–12, and GFAP promoters in all tissues examined. (D) Similar to the promoter library, the normalized expression was scaled for efficiency and specificity by normalizing to the sum across all promoters for each tissue (efficiency) or to the sum across all tissues for each promoter (specificity). This allowed for the comparison of promoter efficiency for each individual tissue (E) and the comparison of tissue specificity for each promoter (F)—in other words, the contribution of expression from each tissue examined.

To confirm these results and to generally assess the predictive value of the library data for the performance of individual promoters, we selected the GFAP promoter and three additional candidates for a subsequent validation experiment (Figure 4). The other promoters were CMV1 representing widely used ubiquitous promoters, as well as SPc5–12 and LP1 as typical synthetic promoters with tissue specificity for muscle or liver, respectively. Each of the four promoter-*eyfp* constructs was packaged in AAV9 and administered to female C57BL/6 mice (*n* = 4 per vector) via tail vein injection (Figure 4A) at a dose of 1 × 10^12^ VG/mouse. Two weeks after injection, vector biodistribution was determined from gDNA by droplet digital (dd)PCR and relative *eyfp* expression from cDNA by qPCR and normalized to the *PolR2A* housekeeper (Figure S9). To compare promoter performance independent of vector biodistribution, a normalized expression value *Qαβ* was calculated for each tissue *β* by normalizing the relative expression *Cβ* [2^−ΔCt^] to the vector biodistribution *Gβ* (VG per diploid genome [DG]) (Figure 4B).

For each of the 4 constructs, *eyfp* expression and thus promoter activity was detected in all 16 tissue types (Figure 4C). The normalized expression profiles were unique and differed between the promoters, with CMV1 performing best in pancreas, gut, liver, and heart; LP1 in liver; SPc5–12 in liver, heart, and skeletal muscle; and GFAP in liver. The profiles observed for the individual constructs largely mirrored those from the initial promoter library screen (Figures 2, 3, and S10).

**Figure 5.**
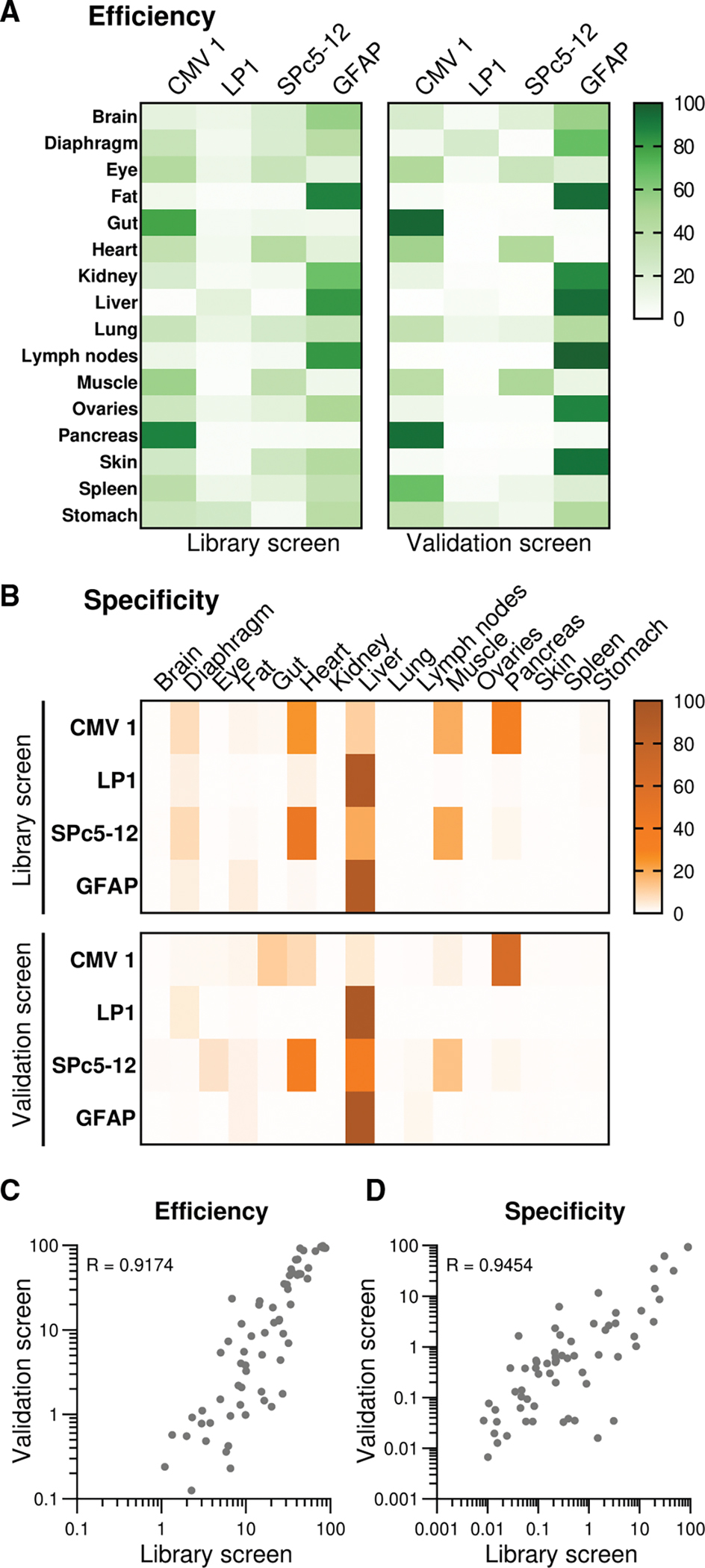
Correlation of expression levels between the initial library screen and the single-promoter validation experiment (A and B) Efficiency (A) and specificity (B) scores from the promoter library experiment were extracted for the CMV1, LP1, SPc5–12, and GFAP promoters. They were then compared to the corresponding scores derived from the single-promoter experiment. Shown are heatmaps of efficiency and specificity scores averaged from six animals for the library screen or from four animals per promoter for the validation screen. (C and D) Correlation of matched average efficiency and specificity scores for each tissue measured with the promoter library or individually injected constructs for the CMV1, LP1, SPc5–12, and GFAP promoters. Pearson correlation coefficients R were calculated using GraphPad Prism.

Next, we calculated efficiency and specificity scores for the validation dataset based on the *Qαβ* values (Figure 4D), similar to the normalization strategy we used for the library data (Figure 1C). For the GFAP promoter, this confirmed both superior efficiency (Figure 4E) for hepatic expression and a liver-directed specificity comparable to the LP1 promoter (Figure 4F). To allow for an even more direct comparison of the screening and validation results, data for the CMV1, LP1, SPc5–12, and GFAP promoters was extracted from the library dataset to calculate efficiency and specificity scores for this subset. This resulted in two equivalent sets of efficiency and specificity scores across all 16 tissues, one derived from the full library screen and the other from the validation screen (Figure 5). A comparison of the average efficiency scores showed that the library screen had correctly identified the strongest promoter in 14 of the 16 tissues evaluated (Figure 5A), with marginal differences in heart (best in library screen: SPc5–12; best in validation screen: CMV1) and muscle (best in library screen: CMV1; best in validation screen: SPc5–12). Regarding tissue specificity, the library screen had implied the strongest expression of the CMV1 promoter in pancreas, LP1 in liver, SPc5–12 in heart, and GFAP in liver (Figure 5B, top). This result was consistent with the top specificity scores in the validation screen (Figure 5B, bottom), except for SPc5–12, whose normalized expression was slightly higher in the liver than in the heart. Overall, the direct comparison of the library and validation data showed a strong correlation for both efficiency (Figure 5C) and specificity scores (Figure 5D), validating our screening pipeline.

The strong *eyfp* expression from the human GFAP promoter in AAV9 that we detected in the murine liver prompted us to ask whether the endogenous mouse *Gfap* gene is expressed in the liver as well. However, a qPCR analysis of endogenous *Gfap* expression compared to the *PolR2A* housekeeper showed the highest values for brain and eye, while other tissues including liver showed only minimal expression (Figure S11A). A possible explanation for the latter may be epigenetic silencing. Publicly available bisulfite sequencing data from murine liver samples (SRR24770973 and SRR24770974) shows methylation within the endogenous mouse *gfap* promoter and coding sequence, yet close to none near the transcription start site (Figure S11B). Interestingly, this pattern is reminiscent of highly transcribed genes,^53^ which stands in contrast to the qPCR results above. This discrepancy suggests that the epigenetic *Gfap* silencing in the murine liver does not occur through DNA methylation but rather other mechanisms such as histone post-translational modifications.^54^ To investigate DNA methylation for the CMV, LP1, and GFAP promoter-bearing AAV constructs employed in our validation screen, we performed enzymatic methylation sequencing (EM-seq) on livers from animals treated with these vectors (Figure S11C). This analysis showed only minimal levels of CpG methylation within the AAV vector sequences, which is in line with the robust reporter expression observed in these mice.

### The GFAP promoter displays hepatocyte specificity in the AAV9 context

**Figure 6.**
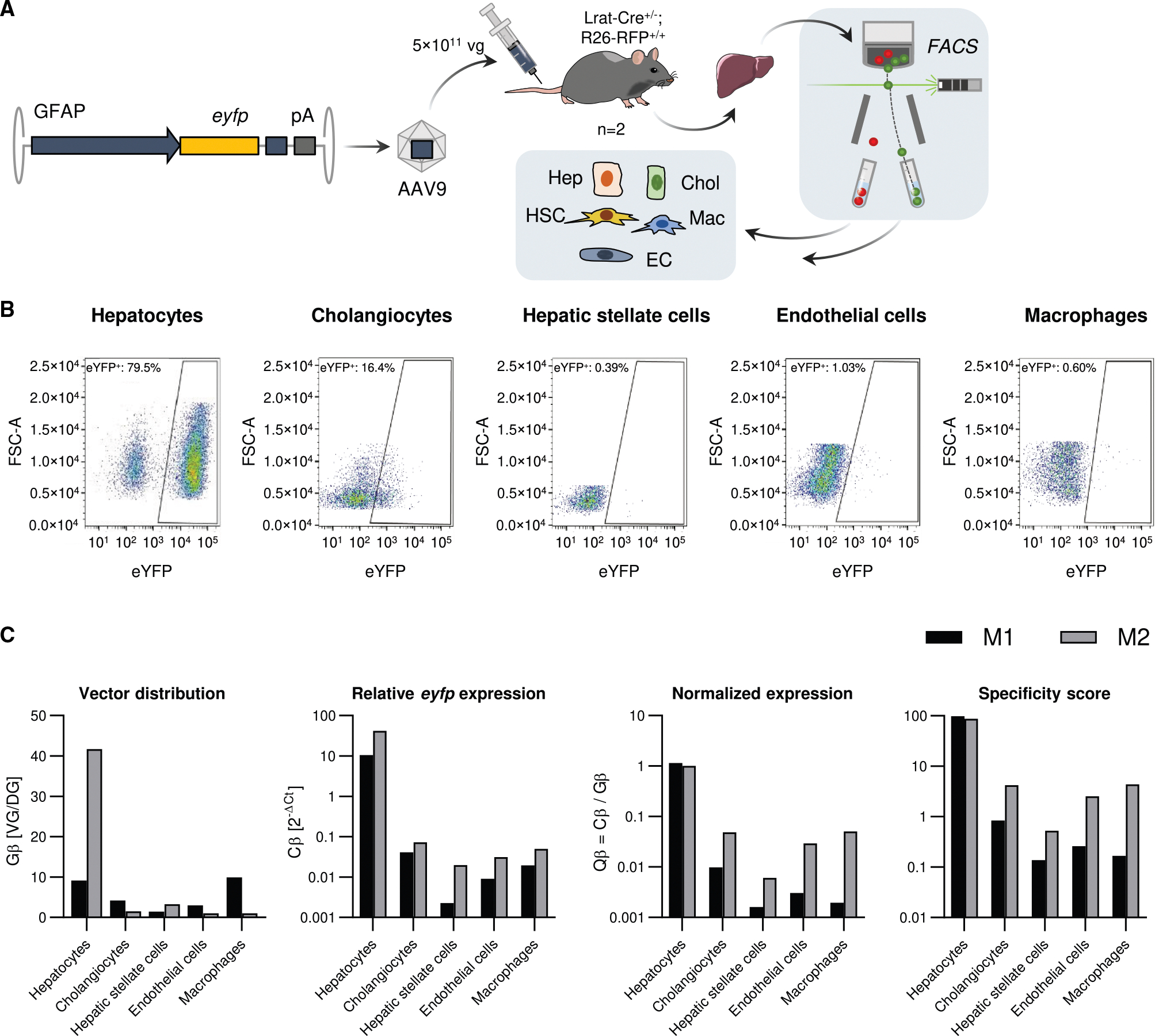
GFAP promoter results in robust expression in mouse hepatocytes (A) The GFAP-*eyfp* construct was packaged into the AAV9 capsid and systemically delivered to two female *Lrat-Cre*^*+/*−^ *;R26-RFP*^+/+^ mice (M1 and M2) at a dose of 5 × 10^11^ VG/mouse. Mice were analyzed 2 weeks after injection, including FACS-based isolation of hepatocytes, cholangiocytes, hepatic stellate cells (HSCs), endothelial cells, and macrophages, as well as immunofluorescence of tissue sections. (B) Flow cytometry of major liver cell types for mouse M2 according to (A). (C) DNA and RNA were extracted from the different cell populations of the two mice (M1 and M2) to measure vector distribution from gDNA by ddPCR (*Gβ*) and relative *eyfp* expression from cDNA by qPCR (*Cβ*). Normalized expression and specificity scores were calculated from *Gβ* and *Cβ*.

The consistent finding in the library and validation screens of superior *in vivo* GFAP promoter activity in the mouse liver motivated us to study the contribution of the different liver cell types to transgene expression from this promoter. For this, we delivered 5 × 10^11^ VGs of the AAV9-GFAP-*eyfp* vector by retro-orbital systemic injection to *Lrat-Cre*^*+/*−^ *;R26-RFP*^+/+^ mice (*n* = 2), followed by isolation of the primary liver cell types by fluorescence-activated cell sorting (FACS) 2 weeks later (Figure 6A). Quantification of EYFP^+^ cells for mouse M2 showed the highest transduction of hepatocytes (79.5% EYFP^+^ cells), followed by cholangiocytes (16.4%) (Figure 6B). Hepatic stellate cells (HSCs), endothelial cells, and macrophages showed only minimal transduction (0.39%, 1.03%, and 0.60%, respectively). As hepatocytes make up 70% of all liver cells,^55^ the strong performance of the GFAP promoter in these cells explains the results in the bulk liver samples (Figures 2 and 4).

**Figure 7.**
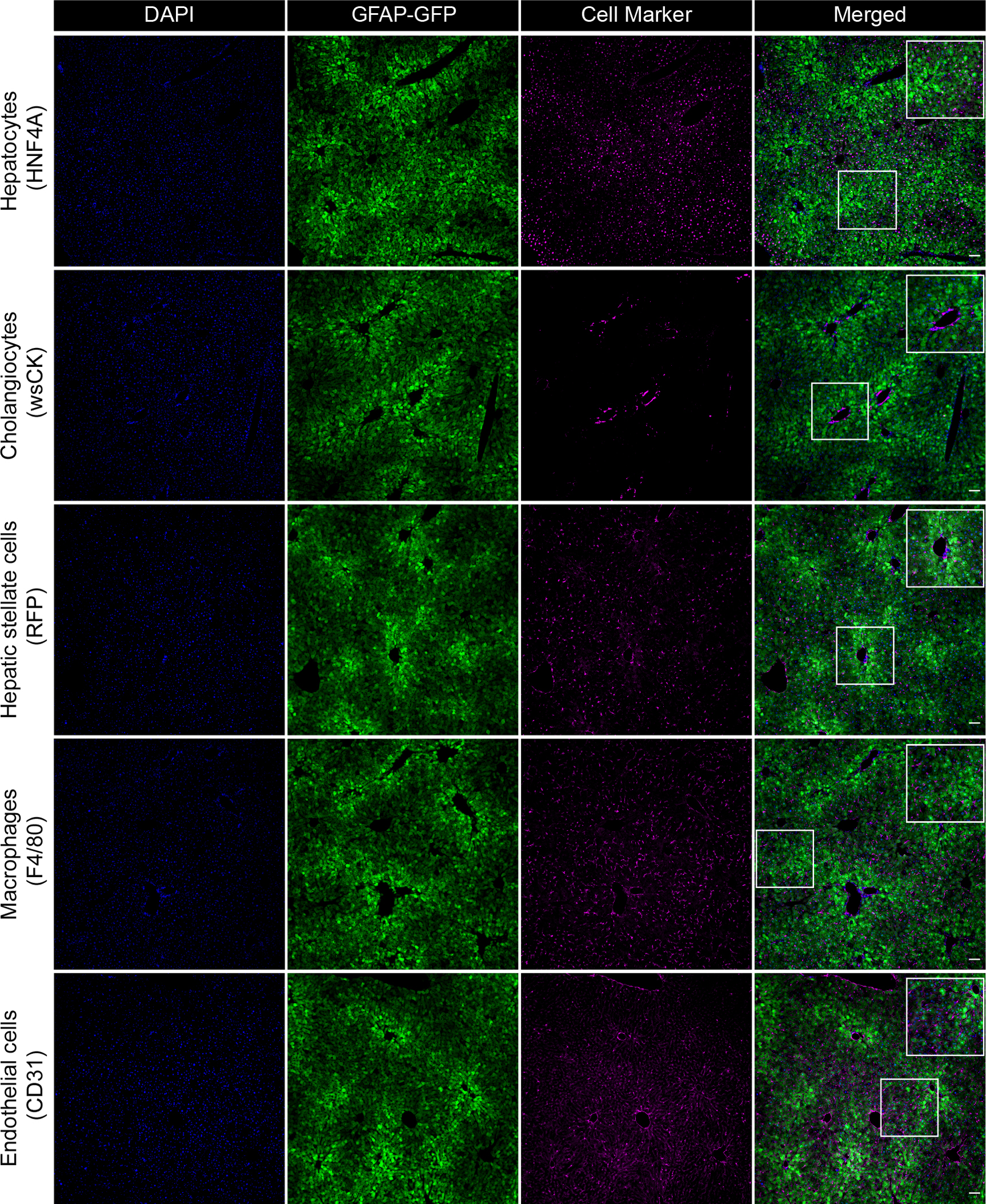
Widespread GFAP promoter-driven EYFP expression in mouse hepatocytes Liver sections from *Lrat-Cre*^*+/*−^ *;R26-RFP*^+/+^ mouse injected with 5 × 10^11^ VG of AAV9-GFAP-EYFP were analyzed by immunofluorescence for EYFP and indicated cell-type-specific markers (mouse M2 from Figure 6). HSCs were identified by immunofluorescence for the fluorescent reporter (dsRed). Scale bars represent 50 μm.

To investigate the possible effect of the cellular tropism of the AAV9 capsid on these results, we measured vector presence and transgene expression in the different liver cell types by extracting DNA and RNA from the FACS-sorted cell populations. As before, VG distribution (*Gβ*) was measured by ddPCR from gDNA and relative *eyfp* transgene expression (*Cβ*) by qPCR from cDNA (Figure 6C). This allowed us to calculate normalized expression (*Qβ*) and specificity scores and, in turn, to examine promoter specificity in the various cell types in both replicate mice. Despite differences in vector copy number in the different liver cell populations between the two mice, the highest relative *eyfp* expression was consistently detected in hepatocytes, followed by cholangiocytes. Normalized expression and specificity scores matched this trend, with a clear and consistent specificity for hepatocyte-directed expression in both mice. These results were further corroborated by immunofluorescence microscopy analysis of liver sections from mouse M2 (Figure 7), which again showed robust and widespread EYFP signals throughout all hepatocytes.

### Functional dissection of the GFAP promoter and validation in a humanized liver mouse

**Figure 8.**
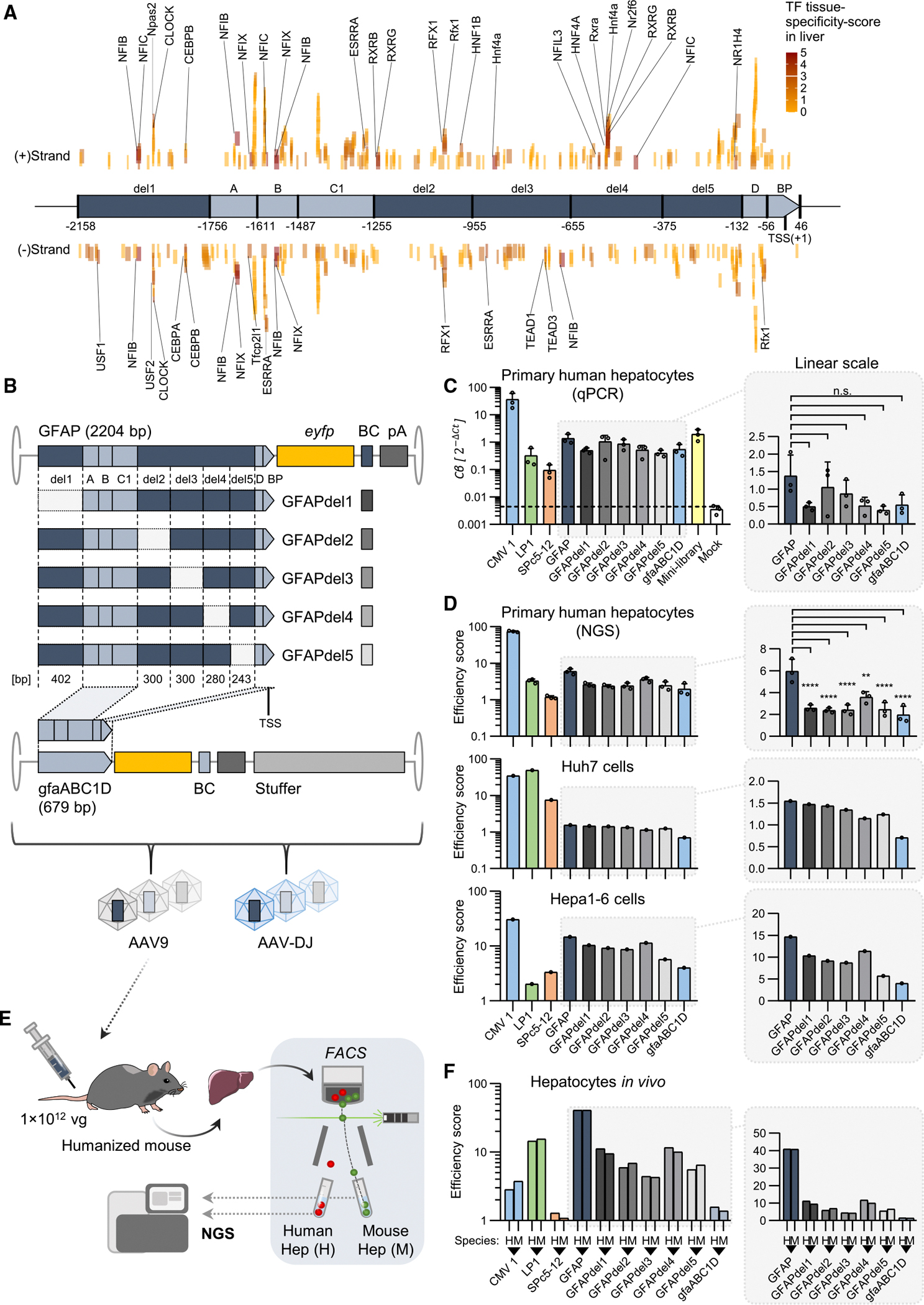
Dissection of GFAP promoter activity in cultured cells and primary hepatocytes (A) Predicted binding sites for hepatocyte-specific transcription factors within the GFAP promoter sequence (liver tissue-specificity-score from Zhou et al.^56^). Based on the comparison with gfaABC1D, five GFAP-unique regions (del1–5) were selected for stepwise deletion. (B) Construction of GFAP deletion variants compared to full-length GFAP and gfaABC1D promoters. GFAP promoter variants and control promoters (CMV1, LP1, SPc5–12) were packaged into AAV9 or AAV-DJ to generate barcoded mini-libraries. (C) Relative *eyfp* expression levels measured by qPCR in primary human hepatocytes transduced with the individual promoter constructs. (D) Primary human hepatocytes, Huh7 cells (human hepatoma), and Hepa1–6 cells (mouse hepatoma) were transduced with the AAV-DJ mini-library (MOI 1 × 10^5^). DNA and RNA were extracted on day 3 post-transduction, followed by barcode readout on the DNA and RNA levels. Efficiency scores were calculated as described in Figure S1. (E and F) The AAV9 promoter mini-library was used for transduction of a liver-humanized FRGN mouse. At 1 week after retro-orbital injection, the mouse was sacrificed for liver harvest and FACS-based isolation of human and mouse hepatocytes. (F) DNA and RNA were extracted from isolated hepatocytes of both species for barcode readout. Efficiency scores were calculated as before. For the GFAP promoter and derivatives, the relative expression (C) and efficiency scores (D and F) were isolated and plotted on a linear scale for better comparison. Statistical analyses between GFAP and derivatives were conducted using ordinary one-way ANOVA with Dunnett’s multiple comparison test (n.s., not significant; *p < 0.05; **p < 0.01; ***p < 0.001; ****p < 0.0001).

A curious observation with our original library was that the gfaABC1D promoter,^49^ a truncated version of the GFAP promoter (679 versus 2,204 bp), was substantially less active in the mouse liver (Figures 2A and S3). In an effort to delineate the regions in the full-length GFAP promoter responsible for its superior activity, we identified putative hepatocyte-specific transcription factor binding sites (TFBS; Figure 8A) and then used this information to generate the five GFAP promoter deletion mutants shown in Figure 8B. They were next produced together with the parental GFAP, gfaABC1D, CMV, LP1, and SPc5–12 promoters as barcoded vectors in the context of an AAV9 or AAV-DJ capsid, resulting in 2 homogeneous libraries of 10 promoter variants each (Figure S12A). While AAV9 was chosen for its high efficiency *in vivo*, AAV-DJ was selected for *in vitro* tests due to its broad activity and high efficiency in multiple cultured and primary cell types.^57^

We used the separate promoter constructs in AAV-DJ as well as the AAV-DJ mini-library to transduce primary human hepatocytes. In the single-promoter experiment (Figure 8C; MOI 1 × 10^5^), analysis of *eyfp* mRNA expression using qPCR revealed a striking contrast to the mouse liver data, as the CMV promoter outperformed all tested promoters including GFAP *in vitro*. Due to sample variation, we could not observe statistically significant differences in the expression of full-length GFAP compared to its derivates GFAPdel1–5 and gfaABC1D. For a more sensitive readout compared to qPCR, gDNA and cDNA of the cells transduced with the AAV-DJ mini library were subjected to NGS analysis of promoter-associated barcodes as was done before for the promoter library *in vivo*. The results (Figure 8D, top) were highly similar to those obtained with the individual constructs, as CMV outperformed all other promoters, followed by full-length GFAP. GFAP derivatives yielded significantly lower efficiency scores as compared to the full-length promoter, whereas the deletion mutants performed between GFAP and the short gfaABC1D promoter. This indicates that each deleted region contributes to the activity of the full-length GFAP promoter. Results were similar in Hepa1–6 (mouse) and Huh7 (human) liver cell lines transduced with the AAV-DJ library (Figure 8D), where CMV1 outperformed most of the other promoters including GFAP. The LP1 and SPc5–12 promoters showed strong activity in Huh7 cells, while they were weaker in primary human hepatocytes and Hep1–6 cells. Notably, LP1 was stronger than GFAP in Huh7 cells, in contrast to its inferior performance in primary human hepatocytes (Figures 8C and 8D) and mouse livers (Figure 2). To validate the robustness of the NGS-based readout of promoter activity through their associated barcodes, we transduced Hepa1–6 cells with different MOIs of the AAV-DJ library ranging from 10^3^ to 10^6^. The fact that we measured only minor differences in transduction efficiency confirms a high independence of the assay of vector doses (Figure S12B).

Because of the marked differences in GFAP promoter activity relative to benchmark promoters in cultured cells versus mouse liver, we investigated species-specific and *in vivo* effects by injecting the AAV9 library containing the 10 aforementioned promoters into an FRGN (*Fah*^−/−^ *;Rag2*^−/−^ *;Il2rg ;Sirpa*^*NOD/NOD*^) mouse repopulated with primary human hepatocytes (Figure 8E). FACS-based isolation of human and mouse hepatocytes, as well as quantification of promoter activity by barcode sequencing, enabled several important conclusions (Figure 8F). First, all promoters tested performed almost identically in mouse and human hepatocytes. Second, consistent with our initial *in vivo* data, the GFAP promoter outperformed all others, including CMV (which was more efficient in all cultured cells, see above), once again illustrating the often pronounced differences between *ex vivo* and *in vivo* AAV vector performance. Third, along the same lines, the muscle-specific SPc5–12 promoter had low activity in human and mouse hepatocytes, in contrast to cultured cells where it was active and even better than LP1 (Hepa1–6) or GFAP and all variants thereof (Huh7). Finally, the differences in performance between full-length GFAP, the minimal gfaABC1D, and the five intermediate mutants were more pronounced in the humanized liver mouse than in cultured cells. In particular, deletion mutants 1 and 4 were more active than mutants 2, 3, and 5, suggesting that the latter three, as well as their TFBSs, may be particularly important *in vivo*.

## DISCUSSION

Since the discovery that natural AAV isolates exhibit distinct clinically relevant properties, major efforts in the field have centered on the design and use of high-throughput technologies for AAV capsid diversification and for massively parallel *in vivo* screening of the resulting libraries.^6,7,58^ Concomitantly, a multitude of techniques was devised to permit additional control over vector efficiency and specificity on other levels, with transcriptional control through promoter selection and engineering perhaps offering the greatest potential and flexibility.^10,18,59,60^ These synergistic efforts were driven by the realization that even highly engineered capsids often fail to mediate the desired level of target organ or cell specificity without additional fine-tuning beyond cell attachment and entry.^37,61^ Moreover, increasing evidence implies a role for the viral capsid beyond delivery of the encoded recombinant DNA, specifically an interaction with encapsidated promoter sequences that impacts transcriptional activity and that necessitates a better understanding of the underlying mechanisms.^62,63^ Finally, the recent advent of methodologies for AAV capsid library selection on the viral mRNA level^36,64,65^ motivates efforts to harness tissue- or cell-specific promoters for expression of engineered *cap* sequences that concurrently function in HEK293 producer cells, which further stimulates basic research into promoter biology in an AAV context.

In this study, we have implemented and validated a pipeline enabling parallel *in vivo* screening of a multitude of barcoded promoter variants within a given AAV capsid (here, AAV9), based on lessons learned and tools established in prior studies by us and others. Akin to the rationale underlying barcoding of capsid libraries, the ability to assess and compare a set of promoters in the same animal offers several benefits, including a reduction in animal numbers as well as in associated labor, time, and costs, combined with increased reproducibility due to the elimination of inter-animal variation. Moreover, this multiplexed approach permits a much higher throughput as compared to studies in which selected promoter (or enhancer) variants were tested separately in individual animals, which limited the number of sequences that could be studied and hence diminished success rates. Nonetheless, these low-throughput screens yielded pivotal insights into promoter functionality and identified relevant candidates for various applications. Examples include a study by Kurosaki et al. in which two promoters were screened in nine different AAV capsids, revealing superior performance of the AAV6-CAG combination in the mouse lung.^66^ Another representative example from the Chuah and VandenDriessche group identified robust *cis*-regulatory modules (CRMs) in the mouse muscle or liver following individual tests of selected CRMs by hydrodynamic tail vein injection or systemic AAV9-mediated delivery.^67^ Lastly, as noted, Jüttner et al. showed the capacity of such screens by concurrently testing 230 promoter variants in 3 different AAV capsids in mice, although this required a significant and exceptional effort.^12^

Regardless of throughput, a seminal aim during promoter library design is always the ability to qualitatively and quantitatively track all variants on the DNA and mRNA levels. In our own design, this was achieved by harnessing constructs we had previously employed in AAV capsid library screens, encoding an *eyfp* reporter tagged with a promoter-specific barcode in its 3′ UTR. This is also one of the two designs that is frequently employed in MPRA screening technologies such as self-transcribing active regulatory region sequencing, the paralleled enhancer single-cell assay, and others.^30–33,68^ Promoter barcoding in AAV has also been reported recently by others, including Westhaus et al., who compared the ability of the AAV p40 promoter to drive the expression of *cap* gene libraries to other known promoters.^36^ A second, more recent example is exciting work from the Davidson laboratory in which barcoded promoter libraries were screened in AAV2 or AAV4 for optimal candidates in the ependyma of mice or non-human primates.^35^

While sharing the overall concept, the library and results reported here are distinguished by several means, most notably the fact that we studied a more comprehensive collection of over 50 promoter variants across 16 tissues and that our primary aim was to generally characterize the power and potential of this high-throughput technique, rather than screening for a distinct candidate for a given application. To this end, and to enable promoter screening in different tissues, we systemically delivered our library using the AAV9 capsid that is known for its broad transduction profile, including efficient targeting of the heart, liver, skeletal muscle, and brain in mice.^37,39^ As was hoped for, we could detect our vectors on both the DNA and RNA levels across all 16 tissues tested. It was further reassuring to find a strong correlation of the data obtained with the high-throughput barcoded promoter library screen with the results from our validation screen using conventional low-throughput, single-construct testing by qPCR. This correlation validated the approach and diminished concerns about promoter interference—in other words, competition for limiting cellular TFs that could have biased the results in the library context. Further validation of our pipeline was provided by the finding that our promoter library dataset confirms many previously reported expression profiles, including the ubiquitous nature of the CAG promoter^14,35,36^ and the high activity of the SPc5–12 promoter in the heart and skeletal muscle^48,69^ of the troponin T (TnT) promoter in the heart^70,71^ and of the LP1 promoter in the liver.^25^

At the same time, even promoters with presumably limited activity in the liver, such as SPc5–12 and TnT,^48,70^ yielded noticeable off-target expression in this tissue. For SPc5–12, we also observed hepatic expression in the single-construct validation screen, indicating that this is not an artifact of the library screen but rather related to the AAV vector context. Interestingly, AAV inverted terminal repeats (ITRs) and flanking regions were reported to mediate the binding of liver-specific TFs and induction of transcription.^72^ It is thus tempting to speculate that this binding contributes to an altered promoter expression profile in an AAV context and leads to the background expression observed in the liver. Specifically, a binding site for the liver-specific TF hepatocyte nuclear factor-1α (HNF-1α) is located next to the AAV2 ITR and is thus also present in our constructs, where it flanks each promoter. This site was described by Logan et al. to infer hepatocyte-specific expression *in vitro* and *in vivo*, which may explain the hepatic activity observed for most of our promoter constructs.^72^ Ideally, future barcoded promoter screens should thus include a negative control lacking any enhancer/promoter sequence to observe the background activity of ITR-induced expression in the liver and elsewhere. Alternatively, to overcome unwanted expression in the liver, the HNF-1α binding site can be mutated.^72^ In case the use of a promoter in an AAV vector context requires additional downregulation of expression in the liver or other tissues, the 3′ UTR of the transgene can be engineered to carry miRNA-based on-switches that allow for fine-tuning of expression, such as miR-122 in the liver.^17–19,73^ Notably, the design of these off-switches is compatible with the barcoding approach employed in our study; hence, the latter can also be exploited to screen for optimal miRNA-binding sites, either alone or in combination with promoters and/or capsids.

Besides the independent confirmation of expected promoter activities, a remarkable and most surprising result of our work is the identification of the high specificity and high efficiency of the full-length GFAP promoter in the liver and, more specifically, in hepatocytes of mouse or human origin. Typically, endogenous GFAP is known as an expression marker for astrocytes in the CNS and for activated HSCs.^74,75^ Therefore, the 2.2-kb human GFAP promoter is often used in AAV vectors to promote astrocyte-specific transgene expression.^76–78^ In contrast, our use of systemic AAV9-based delivery yielded a different expression profile for the GFAP promoter as we found it to be more active in the liver as compared to any other tissue in both the bulk and individual assays. Furthermore, it outperformed all other tested promoters in the liver, including the synthetic, robust and also hepatocyte-specific LP1.^25^ While GFAP promoter-driven off-targeting after systemic AAV8-based delivery was noted before,^79^ to the best of our knowledge, a liver- and in fact hepatocyte-specific expression profile has not been reported so far for this promoter.

Generally, the notion of differential expression profiles of endogenous versus AAV-encoded regulatory elements is not uncommon and was, for instance, made for CREs as well.^33^ A possible explanation is epigenetic silencing of endogenous promoters, which is absent (or delayed) for exogenously and transiently introduced expression cassettes. This hypothesis is supported by published data suggesting methylation of the endogenous mouse GFAP promoter and by our experimental EM-seq data indicating minimal CpG methylation once this promoter is delivered by AAVs. Endogenous promoter activities may be further governed by flanking or remote sequences in the cellular genome, which are missing in the limited fragments that can be accommodated in AAV. Finally, the AAV ITRs and embedded TFBSs may additionally influence the specificity and/or efficiency of an adjacent exogenous promoter. In the future, it will be informative to perform long-term studies with barcoded promoter libraries and especially the GFAP promoter, to monitor the ability of these promoters to maintain therapeutically relevant expression levels *in vivo*. This may also aid in investigating potential silencing as was observed with other AAV constructs,^80^ as well as with promoters such as CMV outside of the AAV context.^81^ Another factor that may affect the activity of promoters in the AAV vector context is the state of the VGs. AAV genomes can persist either as episomal monomers, as episomal concatemers, or as (randomly) integrated vectors.^80,82^ Presence in these different states, which is influenced by the employed AAV capsid as well as time after transduction, may differentially influence promoter activity. Irrespective of mechanism, the phenomenon of context-dependent discrepant promoter activities needs to be considered in the future, especially in *in silico* analyses that aim to predict optimal promoters for use in AAV based on endogenous expression profiles. By merely relying on the latter, GFAP would not have emerged as an interesting promoter for liver- and hepatocyte-directed gene expression, highlighting the sustained relevance of experimental screenings.

Our analysis using qPCR and FACS of different liver cell populations identified hepatocytes as the primary source of GFAP promoter-driven expression in the liver. Additionally, our finding that the 2.2-kb sequence outperformed the shorter, 0.7-kb gfaABC1D promoter,^49^ a deletion variant of the full-length GFAP, implies differential binding of hepatocyte-specific TFs. As any deletion that we experimentally introduced to the full-length GFAP promoter to approximate gfaABC1D diminished its expression *in vitro* and *in vivo*, we conclude that multiple hepatocyte-specific TFs bind to dispersed regions present in the full-length promoter and then synergistically boost its activity. While their exact identification was beyond the scope of this study, this is an interesting task for future applications of GFAP promoter-driven transgene expression in the liver for basic research or human gene therapy. The latter is implied by our notion that all GFAP promoter variants tested here performed comparably in human and mouse hepatocytes in the FRGN mouse,^83^ indicating a high chance of transferability of preclinical mouse data on GFAP promoter activity to human patients. Applications in gene therapy are manifold and include the treatment of inherited monogenic disorders such as hemophilia A or B (by overexpression of factor VIII or IX, respectively),^84–87^ Fabry disease,^88^ ornithine transcarbamylase deficiency,^89^ or Crigler-Najjar syndrome.^90,91^ Noteworthy in this context is that two systemic applications of AAV5 for liver-directed expression of human factor VIII or IX received regulatory approval by the US Food and Drug Administration and the European Medicines Agency. Valoctocogene roxaparvovec (Roctavian) uses a 0.25-kb hybrid liver-specific promoter (HLP) for the overexpression of factor VIII,^92,93^ while etranacogene dezaparvovec (Hemgenix) expresses the codon-optimized human factor IX Padua variant from the hepatocyte-specific LP1 promoter.^86^ For the 1.6-kb human factor IX cDNA, the GFAP promoter is an intriguing alternative to LP1 that, based on our data, promises higher efficiencies from lower doses. In turn, this would help to reduce manufacturing costs and improve the safety of gene therapy. The 4.3-kb human factor VIII cDNA (containing a deletion in the B domain) leaves less space for a promoter in light of the AAV packaging limit,^92^ yet the identification of critical TFBSs in the full-length GFAP promoter could enable the rational design of much smaller synthetic variants. As an overly strong hepatic expression of factor VIII has been shown to induce endoplasmic reticulum stress, the selection of an attenuated promoter may even be beneficial in this context.^94^ In cases where large promoters need to be combined with large transgenes, data from us and others highlight the potential of engineered bocaviruses as an alternative to AAV that can cross-package and deliver AAV VGs of up to ∼6.1 kb,^95^ which suffices to combine even the full-length GFAP promoter with larger therapeutic cDNAs.

The tools established and the insights gathered here lay the groundwork that facilitates the future study of numerous other interesting aspects of AAV and promoter biology, including expanded investigations into the interactions of AAV capsids and viral transcription. Limitations of the barcoded promoter readout include the necessity of separate productions for each AAV promoter construct to ensure comparable contribution of each construct, as well as the choice of AAV capsid, which defines the range of transduced tissues and cell types. To this end, it will be informative to use other capsids for the delivery of promoter libraries, such as liver-detargeted and muscle-directed AAVMYO/MyoAAV^37,65^ or CNS-targeted AAV9 peptide-display variants.^64,96^ A better understanding of the capsid-promoter interplay, including epigenetic modification of the viral genome, is essential not only for vector optimization but also to inform the use of tissue- or cell-specific promoters for the expression of *cap* gene libraries.^63,97^ Concomitantly, the spatiotemporal resolution of future screens could be improved by merging promoter barcoding with single-cell RNA-seq analysis and by studying different time points.^32,80,98^ This will shed light on the kinetics of promoter activity and may explain some of the discrepancies often seen *in vitro* and *in vivo*,^99–101^ including the relative differences between the CMV and GFAP promoter noted here. Lastly, it should be rewarding to combine barcoding of promoters (transcription), capsids^37,102,103^ (transduction), and miRNA binding sites (post-transcription) in the same library, as this promises the ability to design gene therapy vectors mediating optimal efficiency, specificity, and safety.

## MATERIALS AND METHODS

### Cloning of promoter library

All promoter-containing AAV vector plasmids were assembled based on an AAV-*eyfp* reporter construct, which was designed to facilitate cloning and minimize differences between the final constructs. To create this commonly used vector backbone, a single-stranded (ss)AAV vector plasmid available in our group (D.G.) and originally derived from pSSV9 was modified.^104^ First, oligonucleotides (Merck KgaA, Darmstadt, Germany) carrying BamHI and HindIII restriction sites (for later insertion of stuffer DNA, see below) and flanked by BglII sites were annealed. For this purpose, equal amounts of complementary forward and reverse oligonucleotides were incubated in NEB2 buffer (New England Biolabs [NEB], Ipswich, MA) at 95°C for 5 min, followed by slow cooling to room temperature. The annealed oligonucleotides were then ligated into the BglII-linearized ssAAV vector plasmid. Second, we harnessed a library of barcoded AAV plasmids containing an *eyfp* transgene, unique 15-nt barcodes, and a bGH polyadenylation signal that we have previously reported.^37^ From this library, the *eyfp*-barcode-bGH fragment was excised by NotI and BglII digestion and subcloned into the ssAAV vector plasmid. Since the sizes of the 53 promoters varied considerably, we inserted a fragment of the *lacZ* cDNA as filler DNA into the 3′ UTR of each construct to ensure comparable overall sizes. Depending on the size of the promoter, fragments of approximately 0.8, 1.2, 1.5, 1.8, 2.0, 2.2, or 2.4 kb were PCR amplified (Phusion Hot Start II polymerase; Thermo Fisher Scientific, Waltham, MA) from the *lacZ* cDNA using oligonucleotides with BamHI and HindIII restriction sites for subsequent cloning into the corresponding barcoded ssAAV vector plasmid. Finally, the selected promoters were cloned upstream of the *eyfp* transgene. To this end, each promoter was PCR amplified with oligonucleotides containing SpeI or NotI restriction sites at their ends. Alternatively, NheI or XbaI sites (leaving SpeI-compatible sticky ends) were used if a SpeI site was already present in the promoter sequence. In the two cases where the promoter already contained an endogenous NotI site (CMVCad5 and CMV.MLC260), a BsmBI site was used to create a NotI-compatible overhang. Plasmids from our own collection or from collaborators were used as sources for the different promoters. Detailed information on all promoters (sequences, size and origin) is given in Table S1. PCR conditions were optimized according to the complexity of the promoters, taking into account parameters such as repetitive sequences and GC-rich regions. Five promoters (CAG, UbiC, CMV1, hDes, and hSyn) were ordered as DNA fragments (GeneArt; Thermo Fisher Scientific). The integrity of the AAV ITRs was confirmed in individual clones by restriction digestion with XmaI, and the promoter and barcode regions of all plasmids were confirmed via Sanger sequencing (Eurofins Genomics; Ebersberg, Germany).

### Creation of GFAP promoter truncation mutants

GFAP promoter deletion variants were cloned by PCR amplification of respective fragments from the GFAP promoter and inserted into the ssAAV vector backbones as described above (*eyfp* transgene, bGH polyA signal, 0.8 kb *lacZ* stuffer). GFAP_del1 was amplified using primers GFAP_del1_f (5′-AGA GCA ACT AGT CCC ACC TCC CTC TCT GTG CTG-3′) and GFAP_r (5′-ACC GGT GCG GCC GCC GAG CAG CGG AGG TGA T-3′). The GFAP_del2–5 variants were cloned by overlap-extension PCR, using primer GFAP_f (5′-AGA GCA ACT AGT CCC ACC TCC CTC TCT GTG CTG-3′) and variant-specific reverse primers (del2_r: 5′-CCT GTG CCT CTC TCC CAG AGT CCC CTC ACC CAT TTG TGT C-3′; del3_r: 5′-CCT GTT CTG TAC CCT CAA GAC CAG GCC TTG ACA GCT CCA C-3′; del4_r: 5′-GTG GGG TGG CTC ATG CTT GTG GCC ACC TCA TTT CCT ACT AGG-3′; del5_r: 5′-CCC AGC TAT GGG GAG AGC TCA ATC CCA GCA CTT TGG GAG G-3′) for amplification of the 5′ fragment. The 3′ fragment was amplified using variant-specific forward primers with matching overhangs (del2_f: 5′-GAC ACA AAT GGG TGA GGG GAC TCT GGG AGA GAG GCA CAG G-3′; del3_f: 5′-GTG GAG CTG TCA AGG CCT GGT CTT GAG GGT ACA GAA CAG G-3′; del4_f: 5′-CCT AGT AGG AAA TGA GGT GGC CAC AAG CAT GAG CCA CCC CAC-3′; del5_f: 5′-CCT CCC AAA GTG CTG GGA TTG AGC TCT CCC CAT AGC TGG G-3′) and the GFAP_r reverse primer. Amplicons were purified by gel electrophoresis and extraction using the QIAquick Gel Extraction Kit (Qiagen, Hilden, Germany), followed by PCR amplification for 15 cycles without primers and 30 subsequent cycles after the addition of GFAP_f and GFAP_r primers (500 nM final concentration).

### AAV production, purification, and titration

To generate the initial AAV promoter library, barcoded AAV9 vectors were individually produced by triple transfection of HEK293T cells as described,^37^ using vector plasmids containing one of the barcoded promoter-*eyfp* cassettes, an adenoviral helper plasmid, and an AAV helper plasmid encoding AAV2 Rep proteins and AAV9 capsid proteins. AAV particles were purified by iodixanol density gradient centrifugation followed by PBS buffer exchange and concentration on an Amicon Ultra-15 (Merck KGaA).^105^ For titration, AAV particles were subjected to alkaline lysis by mixing 10 μL AAV stock with 10 μL Tris-EDTA buffer (Thermo Fisher Scientific) and 20 μL 2M NaOH, followed by incubation at 56°C for 30 min. The reaction was then neutralized by adding 38 μL 1 M HCl. Lysed AAV particles were diluted and titrated with the ddPCR Bio-Rad system according to the manufacturer’s recommendations and as described (Bio-Rad, Hercules, CA).^42^ Next, 1 × 10^11^ VGs per construct were pooled and concentrated using an Amicon Ultra-15. The final library was titrated as described above prior to injection into mice. AAV9 vectors for single-construct application (packaging *eyfp* constructs with CMV1, LP1, SPc5–12, and GFAP promoters) as well as the AAV9 and AAV-DJ mini-libraries were produced following the same protocol.

### Mouse studies

For the initial promoter library screen and the validation setting with individual promoter constructs, 6-week-old female C57BL/6 mice (Janvier Labs, Le Genest-Saint-Isle, France) were housed and handled in accordance with animal protocols 35–9185.81/G-126/14, 35–9185.81/G-89/16, and 35–9185.81/G-26/20 approved by the Regierungspräsidium Karlsruhe (Germany). Mice were injected intravenously via the tail vein with the barcoded AAV library at 1 × 10^11^ VG per promoter per mouse in a total injection volume of 150 μL/mouse (AAVs were diluted in 1× PBS). For individual promoter constructs, mice were injected with 1 × 10^12^ VG of AAV9-CMV1-*eyfp*, AAV9-LP1-*eyfp*, AAV9-SPc512-*eyfp*, or AAV9-GFAP-*eyfp* via tail vein injection. Two weeks post-injection, mice were sacrificed by CO_2_ inhalation. Quickly after death, all relevant organs were harvested, washed briefly in PBS, and tissue pieces were immersed in RNAlater solution (Thermo Fisher Scientific) before storing at −20°C. All procedures involving the use and care of animals were performed in accordance with Directive 2010/63/EU of the European Parliament and the German Animal Welfare Act.

To validate the GFAP-based *eyfp* expression in mouse liver across different cell types, 5 × 10^11^ VG of the AAV9-GFAP-*eyfp* vector were delivered via retro-orbital injection into two *Lrat-Cre*^*+/*−^ *;R26-RFP*^+/+^ mice, in which HSCs are fluorescently labeled.^106^ Due to the robust GFAP promoter-driven *eyfp* expression observed in the livers of *n* = 4 wild-type mice (Figure 4C), we limited this mouse study to *n* = 2. Mice were sacrificed 2 weeks after injection. For analysis of truncated GFAP variants and other promoters in human and murine hepatocytes, and to complement the *in vitro* transduction of primary human hepatocytes, an FRGN mouse transplanted with primary human hepatocytes was injected with 5 × 10^12^ VG of the AAV9 promoter mini-library via retro-orbital injection. In light of the consistently high transduction across mice humanized with hepatocytes from the same donor,^82,107,108^ we tested the mini-promoter library in a single partially liver-repopulated FRGN mouse (4.7% engraftment). All mice were bred and maintained under barrier conditions, and all procedures were approved by the Institutional Animal Care and Use Committee of the University of California, San Francisco.

### *In vitro* transductions

Primary human hepatocytes were kindly provided by the PHH Core Facility of the German Center for Infection Research (DZIF). Primary human hepatocytes and Huh7 and Hepa1–6 cells were each transduced with an MOI of 10^5^ vector copies per cell using the AAV-DJ promoter library. Next to transduction with the library, primary human hepatocytes were also transduced with individual promoter AAV constructs at the same MOI. To assess expression after transduction with different MOIs, Hepa1–6 cells were additionally transduced with MOIs of 10^3^, 10^4^, 10^5^, and 10^6^. Cells were harvested at day 3 post-transduction by trypsinization (0.25% trypsin/EDTA; Thermo Fisher Scientific) and resuspension in DMEM (Thermo Fisher Scientific), followed by washing with PBS and freezing of cell pellets in liquid nitrogen.

### DNA/RNA extraction and cDNA synthesis

For total DNA and RNA extraction from mouse tissues, approximately 10 mg of tissue was homogenized in 350 μL RLT buffer (Qiagen) with 1% β-mercaptoethanol (Sigma-Aldrich, St. Louis, MO) using a TissueLyser LT and stainless steel beads (both Qiagen). Tissue lysates were subsequently centrifuged at 3,200 rcf for 4 min to pellet residual debris. Next, 5PRIME Phase Lock Gel tubes (QuantaBio, Beverly, MA) were centrifuged at 16,000 rcf for 30 s to collect the gel at the bottom of the tube. Then, 400 μL phenol:chloroform:isoamyl alcohol (Merck KGaA) was added. We transferred 400 μL of the cleared tissue lysate to a prepared 5PRIME Phase Lock Gel tube and shaken vigorously for 15 s. After centrifugation at 16,000 rcf for 5 min, 400 μL chloroform:isoamyl alcohol was added, and the tubes were again shaken vigorously for 15 s. Tubes were incubated for 3 min at room temperature before centrifugation at 16,000 rcf for 5 min. Next, 350 μL of the aqueous phase was used to isolate DNA and RNA using the AllPrep DNA/RNA Mini kit (Qiagen). Residual contaminating DNA was removed from the RNA fraction by using the RNase-free DNase I Set (Qiagen; off-column protocol). DNase I was then heat inactivated at 75°C for 10 min. DNA/RNA extraction from FACS-isolated liver cell-type populations was performed using the AllPrep DNA/RNA Micro Kit (Qiagen) according to the manufacturer’s instructions, using QIAshredder columns (Qiagen) for cell lysate homogenization. DNA-free RNA was reverse transcribed to cDNA using the High-Capacity cDNA Reverse Transcription Kit (Thermo Fisher Scientific).

### NGS of amplified barcodes

The barcode region of the cDNA and gDNA samples was amplified by PCR in a 50-μL reaction containing 0.5 μL Phusion Hot Start II DNA Polymerase (Thermo Fisher Scientific), 10 μL 5× Phusion HF buffer, 1 μL deoxynucleotide triphosphates (10 mM stock), 0.25 μL forward primer (5′-ATC ACT CTC GGC ATG GAC GAG C-3′; 100 μM stock), 0.25 μL reverse primer (5′-GGC TGG CAA CTA GAA GGC ACA-3′; 100 μM stock), and 25 ng gDNA or 40 ng cDNA as template. Cycling conditions were 30 s at 98°C, followed by 40 cycles of 98°C for 10 s and 72°C for 20 s, and a final step of 5 min at 72°C. The PCR reaction was subsequently cleaned up with the QIAquick PCR purification kit (Qiagen). To prepare the amplicon sequencing library, the Ovation Library System for Low Complexity Samples Kit (NuGEN/Tecan, Männedorf, Switzerland) was used to process 30 ng amplicon DNA per sample. Results were monitored by running the processed samples on an Agilent 2100 Bioanalyzer using the Agilent DNA 1000 kit (Agilent Technologies, Santa Clara, CA). The DNA concentration of the samples was quantified with the Qubit Fluorometer using the Qubit double-stranded DNA HS buffer (Thermo Fisher Scientific). Based on the DNA concentrations, identical amounts per sample were pooled and each library thus prepared was sequenced at the European Molecular Biology Laboratory (EMBL) GeneCore facility (Heidelberg, Germany). A maximum of 32 samples were multiplexed per flow cell, and a total of 7 flow cells were used for this study. Sequencing was performed on the NextSeq500 system (Illumina, San Diego, CA) using the following instructions: read 1 was set to 84 and index 1–8. A PhiX control was spiked into each library and to balance the base composition.

Stocks and samples derived from the AAV9 and AAV-DJ mini-libraries (10 promoter variants each) were sequenced with the Azenta AmpliconEZ sequencing service (MiSeq) using the primer set AEZ_f (5′-ACA CTC TTT CCC TAC ACG ACG CTC TTC CGA TCT AGT CCG CCC TGA GCA AAG AC-3′) and AEZ_r (5′-GAC TGG AGT TCA GAC GTG TGC TCT TCC GAT CTG GCT GGC AAC TAG AAG GCA C-3′) for amplification of the barcode region.

### Detection of viral genomes by ddPCR

AAV genome copies present in gDNA samples were quantified using the Bio-Rad ddPCR system according to the manufacturer’s recommendations. In a total volume of 20 μL, the PCR reactions contained 2× ddPCR Supermix for probes (no deoxyuridine triphosphate) (Bio-Rad), 20X Rpp30 primer/probe-HEX mix (housekeeper gene, Mmu-Rpp30, Bio-Rad), 125 nM *eyfp* probe (5′-ACG ACG GCA ACT ACA-3′), 900 nM of each *eyfp* primer (forward: 5′-GAG CGC ACC ATC TTC TTC AAG-3′ and reverse: 5′-TGT CGC CCT CGA ACT TCA C-3′), a 4× HindIII enzyme dilution (NEB), and 25 ng input DNA (except for liver samples, where only 2 ng input DNA were used).

Cycling conditions were 10 min at 94°C, 40 cycles repeating 30 s at 94°C and 1 min at 58°C, and finally 10 min at 98°C. After droplet reading (QX200 Droplet reader and QuantaSoftsoftware, Bio-Rad), the resulting Rpp30 values were divided by two to obtain the number of DGs, and *eyfp* copy numbers were divided by the number of DGs. This yielded VGs/DG, here referred to as *Gαβ* (for promoter *α* in tissue *β*).

### Expression analysis by qPCR of cDNA samples

Expression of the *eyfp* transgene, the endogenous *Gfap*, and the *PolR2A* housekeeper was measured from cDNA via qPCR. Reactions were performed using either primer/probe mixes for the detection of *eyfp* mRNA (400 nM of each *eyfp* primer and 100 nM *eyfp* probe per final reaction) or commercially available *Gfap* or *PolR2A* primer/probe mixes (*Gfap*: qMmuCIP0032231, Bio-Rad; *PolR2A:* Mm00839502_m1; Thermo Fisher Scientific). Each 25-μL reaction contained 2× Sensimix II probe kit master mix, 1× primer/probe mix, 500 nM ROX (Bioline, London, UK), and 4 μL cDNA (derived from 15 ng RNA input used in cDNA synthesis). For cDNA derived from primary human hepatocytes, the HPRT primers (forward: 5′-GAG GAT TTG GAA AGG GTG TTT ATT C-3′; reverse: 5′-CTC CCA TCT CCT TCA TCA CAT CTC-3′) and probe (5′-HEX-ACA GGA CTG AAC GTC TTG C-BHQ1–3′) were used for detection of *HPRT* as housekeeper. Each reaction was measured in duplicate using the StepOnePlus Real-Time PCR System (Applied Biosystems/Thermo Fisher Scientific). Relative expression values *Cαβ* were obtained from target (Ct_eyfp_) and housekeeper (Ct_PolR2A_) Ct values by calculating (1) ΔCt, i.e., the difference between Ct_eyfp_ and Ct_PolR2A_, and (2) its fold change (i.e., 2^−ΔCt^). To determine normalized expression values *Qαβ*, relative expression values *Cαβ* were divided by the viral copy number per cell *Gαβ*.

### NGS data normalization

For NGS data processing, we used Python 2.7 scripts established in our group and previously used for barcoded AAV capsid libraries.^42^ To adapt this pipeline for normalization of the data obtained here with the barcoded AAV promoter library, we modified individual steps of the second script. During cloning of the promoter library, each promoter *α* was assigned to a specific DNA barcode located in the 3′ UTR of the expressed *eyfp* transgene. Therefore, NGS of the barcode regions allowed quantification of the proportion of each promoter *α* within different samples. The sequenced samples yielded (1) *Lα*, corresponding to the proportion of each promoter *α* within the injected promoter library; (2) *Pαβ(gDNA)*, corresponding to the proportion of each promoter *α* within the gDNA extracted from organ *β*; and (3) *Pαβ(cDNA)*, corresponding to the proportion of each promoter transcript *α* within the cDNA obtained by reverse transcription of the mRNAs extracted from organ *β* (see also Figure S1). In the next step, the normalized contribution of each promoter *α* to the actual expression within each organ sample *β* was calculated by (1) dividing *Pαβ(cDNA)* by *Pαβ(gDNA)* and (2) multiplying this ratio by the measured relative *eyfp* expression of each organ *Cβ*. This yielded *Rαβ*—in other words, the relative normalized expression of each promoter *α* within each organ *β*.

### Calculation and visualization of efficiency and specificity scores

To compare the efficiency of all promoters within the promoter library in individual tissues, efficiency scores *Eαβ* were derived from *Rαβ* by (1) calculating *Σ*_*β*_*Rαβ* (i.e., the sum of *Rαβ* values from all promoters within each tissue *β* [e.g., brain of mouse 1]), (2) dividing each *Rαβ* value from tissue *β* by *Σ*_*β*_*Rαβ*, and (3) scaling by 100 to obtain scores between 0 and 100. To compare promoter specificity between different organs, specificity scores *Sαβ* were calculated for each promoter α by (1) calculating *Σ*_*α*_*Rαβ* (i.e., the sum of *Rαβ* values from promoter α for all 16 tissues *β*), (2) dividing each *Rαβ* value from promoter α by *Σ*_*α*_*Rαβ*, and (3) scaling by 100 to obtain scores between 0 and 100. To calculate efficiency and specificity scores for the individually injected constructs of the validation experiment (including only CMV1, LP1, SPc5–12, and GFAP), the same calculations as above were performed, but *Qαβ* values (i.e., expression normalized to vector distribution) were used as input. Specificity scores *Sαβ* were calculated for individual mice (*Sαβ* = 100 × *Qαβ*/*Σ*_*β*_*Qαβ*), while efficiency scores *Eαβ* were calculated for each tissue using the mean normalized expression Q*αβ that was averaged across all four replicate mice for each promoter set (*Eαβ* = 100 × Q*αβ/*Σ*_*α*_Q*αβ). Pearson correlation coefficients of efficiency and specificity scores were calculated using GraphPad Prism software (GraphPad, Boston, MA).

To generate heatmaps of efficiency and specificity scores, values were transformed into data matrices to reorder the columns and/or rows according to an unsupervised hierarchical clustering algorithm and rescaled to natural logarithm values to facilitate color scaling in visualization. Clustered gapped heatmaps were generated using the pheatmap package in R. Dimensionality reduction by principal-component analysis (PCA) was based on generated matrix data for each mouse/tissue observation as well as average values for all mice per tissue. PCA was performed using the prcomp function from the stats package in R. Visualizations were performed with the ggplot/2 package in R.

### Tissue preparation for immunofluorescence

Livers from *Lrat-Cre*^*+/*−^ *;R26-RFP*^+/+^ mice injected with AAV9-GFAP-*eyfp* were perfused with ice-cold PBS followed by 4% paraformaldehyde (PFA, VWR International, Radnor, PA). Livers were sliced and fixed in 4% PFA at 4°C overnight. Samples were washed three times for 10 min in cold PBS before transfer to 30% sucrose overnight at 4°C and embedded in Tissue-Tek O.C.T. (Sakura Finetek, Torrance, CA). A Leica CM3050 S cryostat (Leica Biosystems, Wetzlar, Germany) was used to cut 6-μm cryosections for staining and imaging.

### Immunofluorescence microscopy

Cryosections were blocked for 1 h at room temperature in PBT (2.5% BSA, 0.5% Triton X-100 and 0.02% sodium azide in PBS) supplemented with 5% normal donkey serum and then incubated overnight at 4°C in primary antibody diluted in PBT. After five PBS washes of 5 min each, secondary antibodies diluted in PBT were added along with DAPI to stain nuclei. Cells were incubated with secondary antibodies for 1 h at room temperature, followed by five 5-min PBS washes. Sections were mounted in Prolong Diamond Antifade (Molecular Probes, catalog no. P36970; Thermo Fisher Scientific). Antibodies against HNF4A (1:00, catalog no. 3113S, Cell Signaling Technology, Cambridge, UK), CD31 (1:100, catalog no. 550274, BD Biosciences, Franklin Lakes, NJ), F4/80 (1:100, catalog no. 14–4801-82, Thermo Fisher Scientific), wsCK (1:100, catalog no. Z0622, DAKO/Agilent Technologies), dsRed (1:500, catalog no. 632496, Clontech/Takara Bio, Kusatsu, Japan), or GFP (1:1,000, catalog no. ab1397, Abcam, Cambridge, UK) were used with Alexa Fluor-conjugated secondary antibodies (1:500).

### FACS

Mouse livers were enzymatically digested as described previously.^83^ A detailed description of the cell isolation protocol will be published separately. Briefly, viable hepatocytes were collected by centrifuging isolated cells at 50 × *g* for 2 min, followed by Percoll density gradient centrifugation of the pelleted cells. *In vivo* AAV9-GFAP-*eyfp* transduction efficiencies of primary mouse liver cells (hepatocytes, cholangiocytes, HSCs, endothelial cells, and macrophages isolated from the same mouse) were analyzed in *Lrat-Cre*^*+/*−^ *;R26-RFP*^+/+^ mice in which HSCs are fluorescently labeled. All cell types were analyzed for EYFP fluorescence using a BD FACS Aria II, and plots were generated with FlowJo software. For the FRGN mouse repopulated with primary human hepatocytes, isolated hepatocytes were stained with antibodies for non-parenchymal cell markers (mCD45, catalog no. 103133, BioLegend, San Diego, CA; mCD31, catalog no. 102417, BioLegend; mPDGFRB, catalog no. 25–1402-82, Invitrogen/Thermo Fisher Scientific) to exclude contaminating non-parenchymal cells. Species-specific antibodies were used to separate human (hB2M, catalog no. 316305, BioLegend) from mouse (mH-2Kd, catalog no. 116603, BioLegend) hepatocytes. The FACS gating strategy for isolating human and mouse hepatocytes is depicted in Figure S13.

### Prediction of TFBSs in the GFAP promoter

To predict binding sites for hepatocyte-specific TFs in the GFAP promoter, position frequency matrices (PFMs) in MEME format were obtained from the core dataset of human and mouse TFs (depending on availability) from the JASPAR 2022 database.^109^ Next, PFMs were used to scan their occurrences in the GFAP promoter sequence, with *p* values less than 10^−4^ using the FIMO tool (version 5.5.6; https://meme-suite.org/meme/tools/fimo). MEME IDs and annotated TFBS are listed in Table S2, sheet 1. Next, the tissue-specificity scores (TSPS) of respective TFs in the liver were obtained from Zhou et al.^56^ These scores range from 0 (ubiquitous expression of a TF) to 5 (maximum liver specificity). For each TF binding site mapped to GFAP, the respective TSPS from Zhou et al. was annotated (Table S2, sheet 2), and TFs with TSPSs >3 are labeled in Figure 8A.

### EM-seq/bisulfite data generation and analysis

To assess the methylation state of the AAV-delivered episomal DNA, 1 μg extracted liver DNA was digested with exonuclease V (catalog no. M0345S, NEB) to enrich circular episomes before mixing with control DNA and sonication to an expected fragment size of 300 bp (Covaris S2; Covaris, Woburn, MA). End-repair, adapter ligation, oxidation, and deamination were done according to the EM-seq kit protocol (catalog no. E7120, NEB). The final index barcode PCR reaction comprised 12 cycles. Per sample, about 9 million 42-bp paired-end reads were generated by the Illumina MiniSeq system. As these data did not produce sufficient coverage for the assessment of methylation within the mouse genome, we additionally used two control replicates of a publicly available dataset of whole-genome mouse liver bisulfite sequencing (PRJNA977888). For both datasets, adapters were trimmed with Trimmomatic version 0.39,^110^ aligned to the mouse genome (GRCm39) and the viral genomes with bwa-meth version 0.2.7,^111^ and the methylation data were tabulated using MethylDackel version 0.3.0 (https://github.com/dpryan79/MethylDackel).

## Supplementary Material

Figures S1-S13

Table S2

Table S1

Table S3

SUPPLEMENTAL INFORMATION

Supplemental information can be found online at https://doi.org/10.1016/j.ymthe.2025.04.027.

## Data Availability

Raw Illumina sequencing data from the *in vivo* screen of the promoter library have been deposited in the NCBI BioProject: PRJNA1209753. Code for the extraction of *eyfp-*linked barcodes from Illumina libraries can be found in Rapti et al. (script 1).^42^ Parameters for barcode extraction, association with the included promoters, and normalizations can be found in Table S3.
