# Supplementary material for: Identification of a robust promoter in mouse and human hepatocytes by in vivo biopanning of a barcoded AAV library
Source: Mol Ther. 2025 Apr 21;33(8):3881–901. doi: 10.1016/j.ymthe.2025.04.027 (PMC12443165; doi:10.1016/j.ymthe.2025.04.027)
Supplement: Supplementary file 1 [file mmc1.pdf]

**YMTHE, Volume *33***

## Supplemental Information

# Identification of a robust promoter in mouse and human hepatocytes by *in vivo* biopanning of a barcoded AAV library

**Jonas Becker, Claire Domenger, Pervinder Choksi, Chiara Krämer, Conradin Baumgartl, Olena Maiakovska, Jae-Jun Kim, Jonas Weinmann, Georg Huber, Florian Schmidt, Christian Thirion, Oliver J. Müller, Holger Willenbring, and Dirk Grimm**

**Table S1: Detailed information on all 53 promoters included in the primary promoter library.** Information on promoter name, description, supposed tissue specificity, sequence, length, origin and employed stuffer size is provided.

**Table S2: Putative transcription factor binding sites detected within the GFAP promoter.** (Sheet 1) FIMO output for transcription factor MEMEs using the JASPAR Core dataset for all deposited TFs from human and mouse origin[109]. (Sheet 2) Subsection of Sheet 1 with matching transcription factors in the Zhou *et al*. tissue-specificity scores for liver[110].

**Table S3: Parameters used for barcode extraction and NGS analysis of the promoter library dataset.** For more details, see Rapti *et al*. [42].

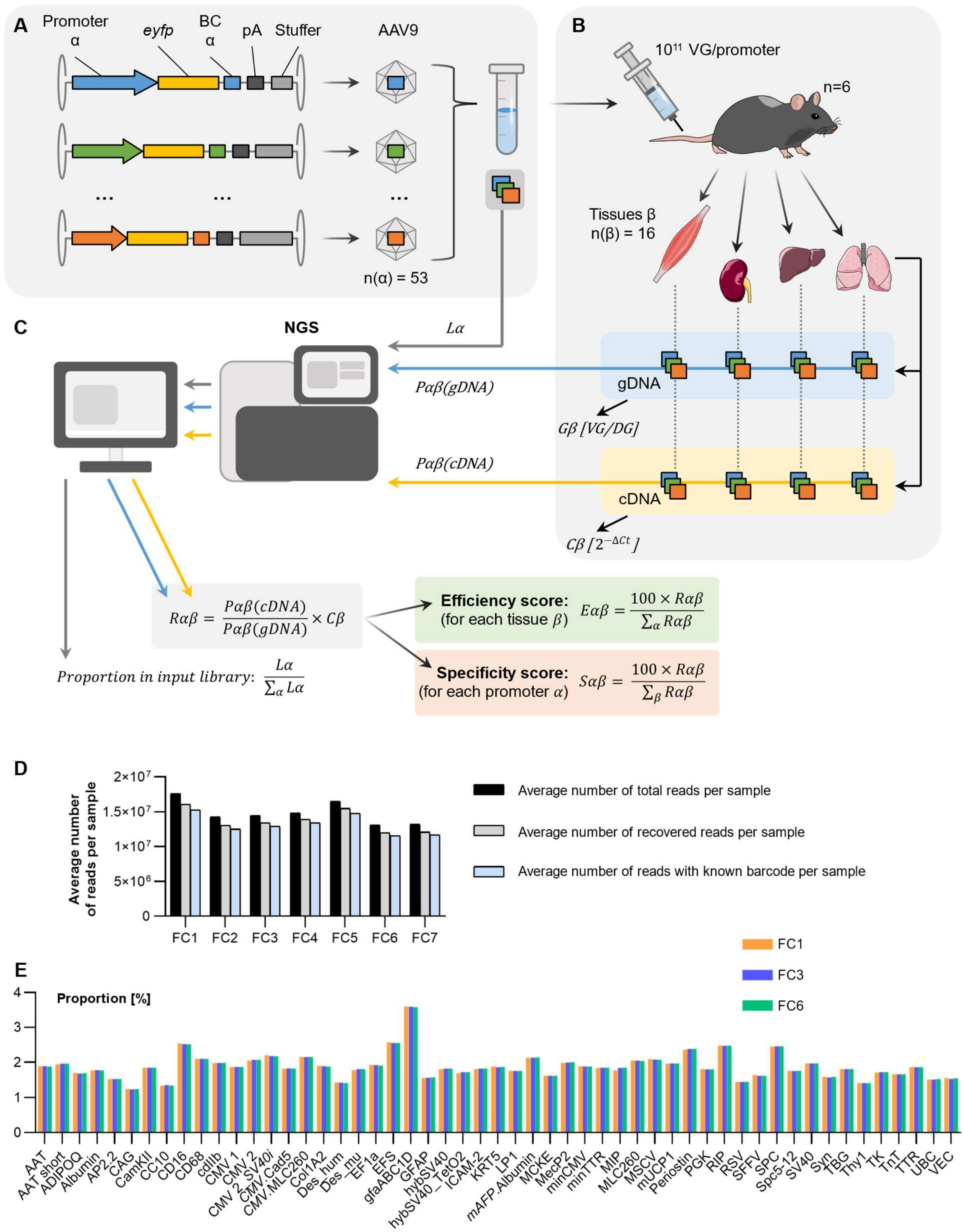


**Figure S1. Detailed normalization strategy and read proportions for the barcoded promoter library screen.** (A-B) A library of 53 different promoters ($\alpha_1$-$\alpha_{53}$) was cloned, produced and

screened in mice as described in Figure 1. Two weeks after injection, 16 different tissues ($\beta_1$-$\beta_{16}$) were harvested and subjected to DNA and RNA extraction. For each tissue *β*, the vector genome copy number per cell was measured by ddPCR from gDNA (*Gβ*), and relative bulk *eyfp* expression was measured by qPCR (*Cβ*). (C) Illumina sequencing was performed to evaluate the contribution of individual promoters *α* to bulk *eyfp* expression. Therefore, barcode-containing amplicons were generated and sequenced to obtain (i) *Lα,* the relative barcode proportions within the input vector library, (ii) *Pαβ(gDNA)*, the barcode proportions in gDNA samples extracted from each tissue *β*, which gives the proportions of individual promoters *α* in tissue *β* at the gDNA level, and (iii) *Pαβ(cDNA)*, the barcode proportions in cDNA samples derived from each tissue *β*, which gives the proportions of individual promoters *α* from each tissue *β* at the cDNA level. To calculate *Rαβ*, the relative normalized expression of each promoter *α* within each organ *β*, *i.e.*, *Pαβ(cDNA),* was divided by *Pαβ(gDNA)* and subsequently multiplied by the relative bulk *eyfp Cβ* of that tissue sample. Finally, *Rαβ* was used to calculate efficiency and specificity scores. For efficiency scores, *Rαβ* values within each tissue sample were normalized to the sum of *Rαβ* values of all 53 promoters within that tissue, followed by scaling by 100 to yield values from 0-100. For specificity scores, *Rαβ* values of a promoter α were normalized to the sum of *Rαβ* across all 16 tissue samples within each replicate animal, also followed by scaling by 100 to yield values from 0-100. (D) Total recovered and correctly assigned read numbers obtained from Illumina sequencing performed in seven flow cells (FC1-7). (E) Barcode proportions of the injected promoter library (Lα) were sequenced three times, with the three samples distributed among flow cells (FC) 1, 3 and 6. Mean values of each barcode fraction were calculated, yielding *Lα*.

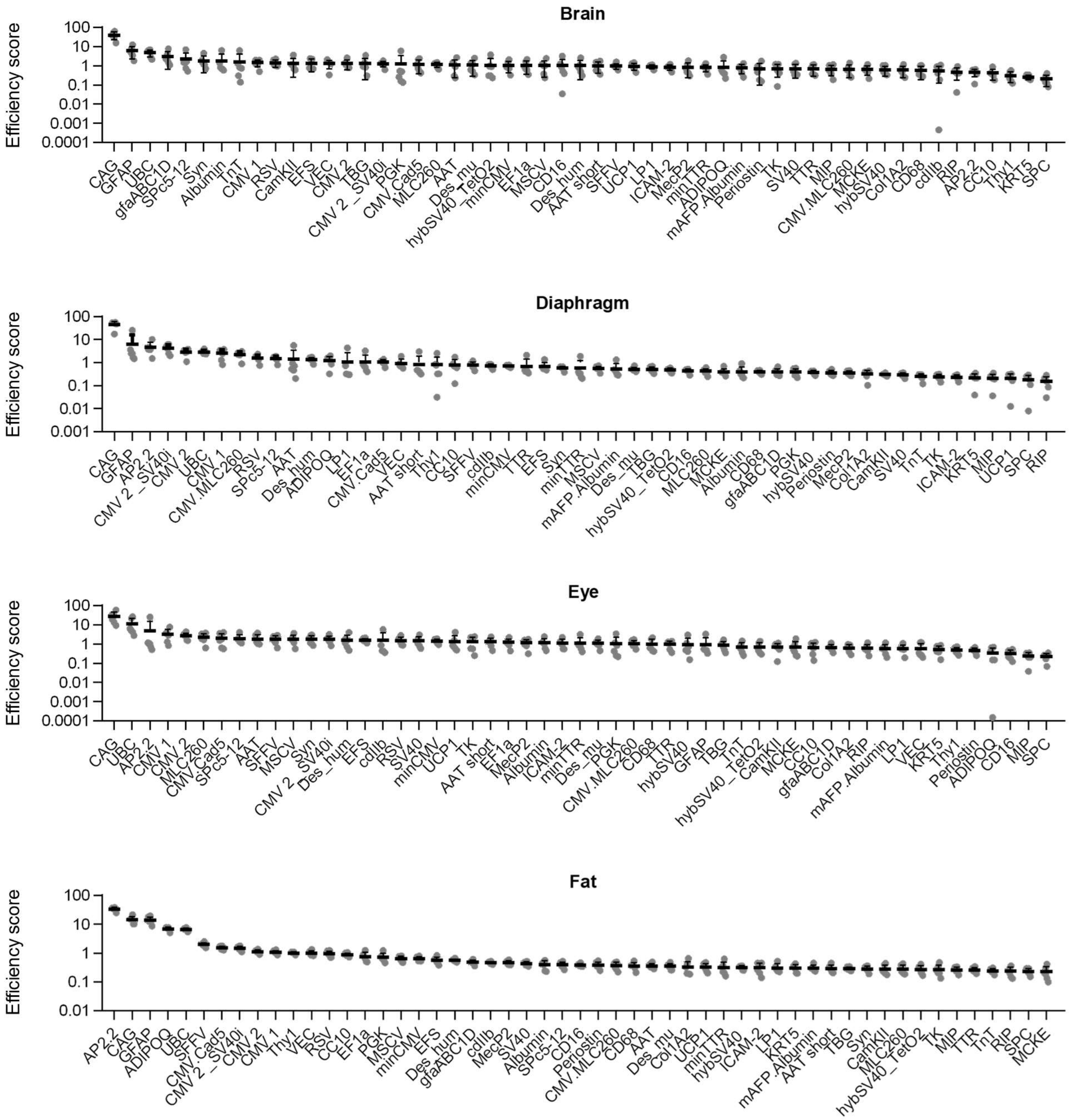


**Figure S2. Ranked efficiency scores of 53 promoters across all 16 tissues examined.** For each tissue sample, efficiency scores were calculated and ranked according to the average score over six replicate animals. Efficiency scores are shown for the following tissues: brain, diaphragm, eye and (white) fat tissue. Replicate values (N=6) are plotted with mean and standard deviation.

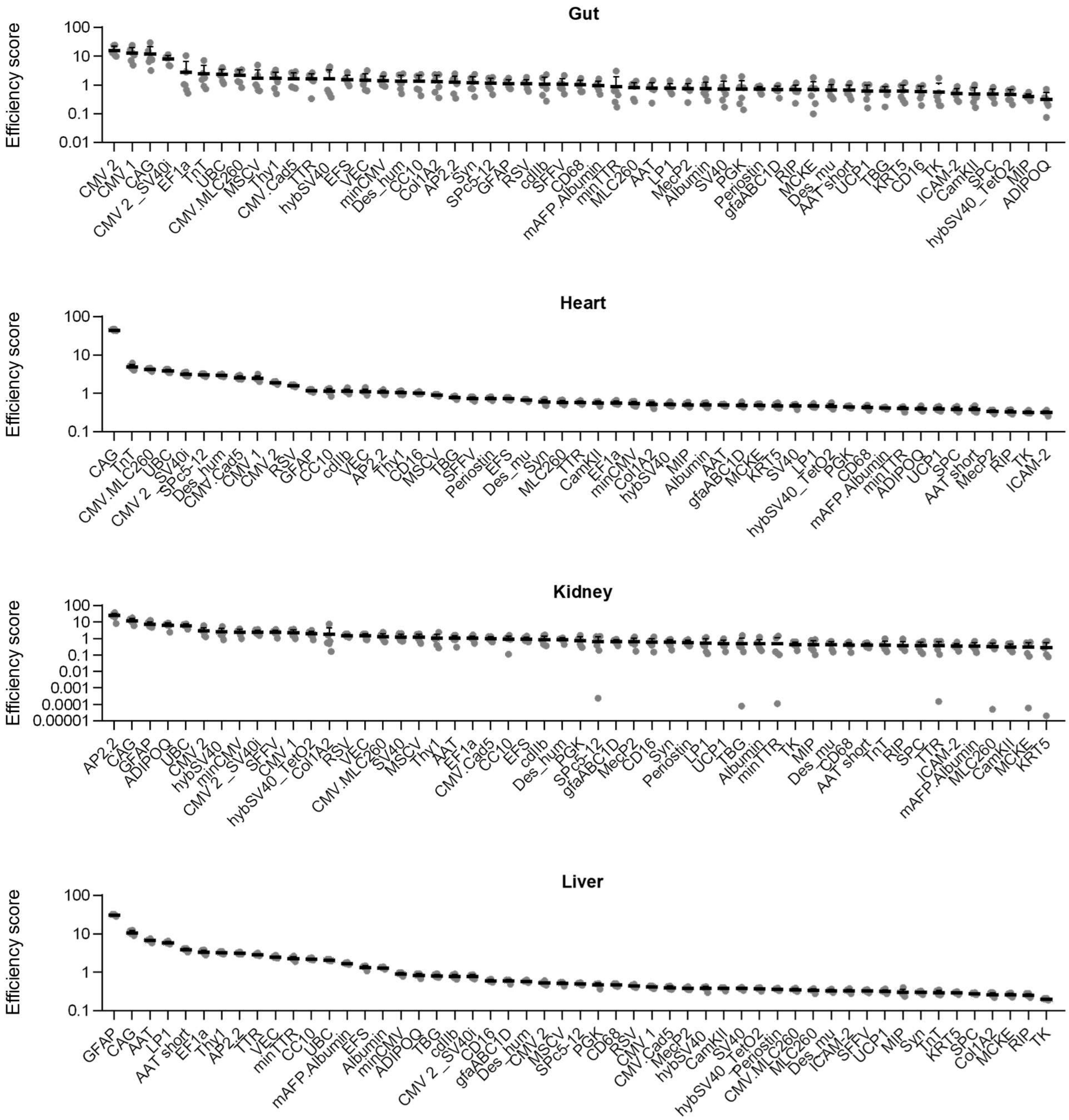


**Figure S3. Ranked efficiency scores of 53 promoters across all 16 tissues examined.** For each tissue sample, efficiency scores were calculated and ranked according to the average score over six replicate animals. Efficiency scores are shown for the following tissues: gut (duodenum), heart, kidney and liver samples. Replicate values (N=6) are plotted with mean and standard deviation.

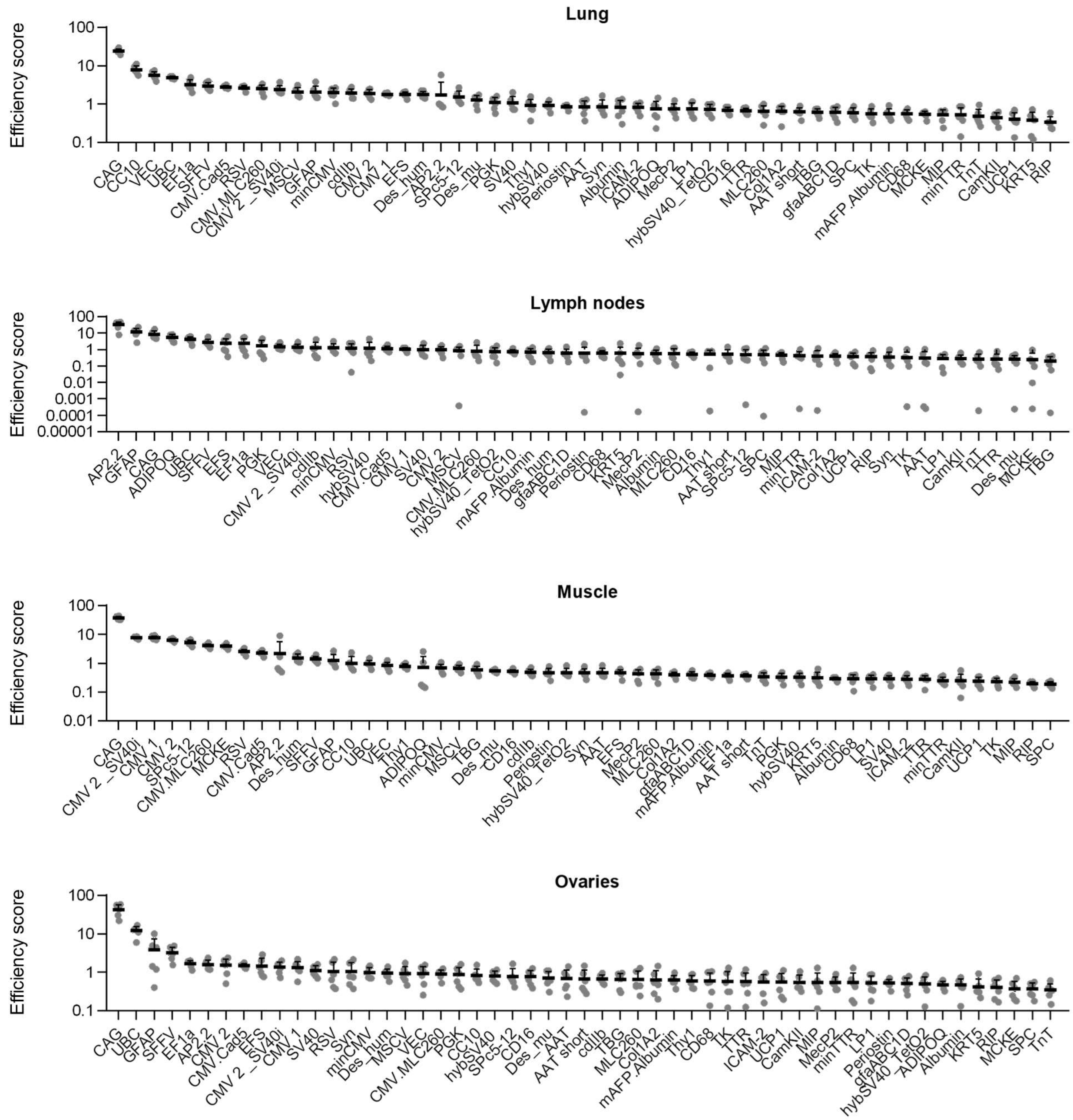


**Figure S4. Ranked efficiency scores of 53 promoters across all 16 tissues examined.** For each tissue sample, efficiency scores were calculated and ranked according to the average score over six replicate animals. Efficiency scores are shown for the following tissues: lung, lymph nodes (inguinal), (skeletal) muscle (*quadriceps femoris*) and ovaries. Replicate values (N=6) are plotted with mean and standard deviation.

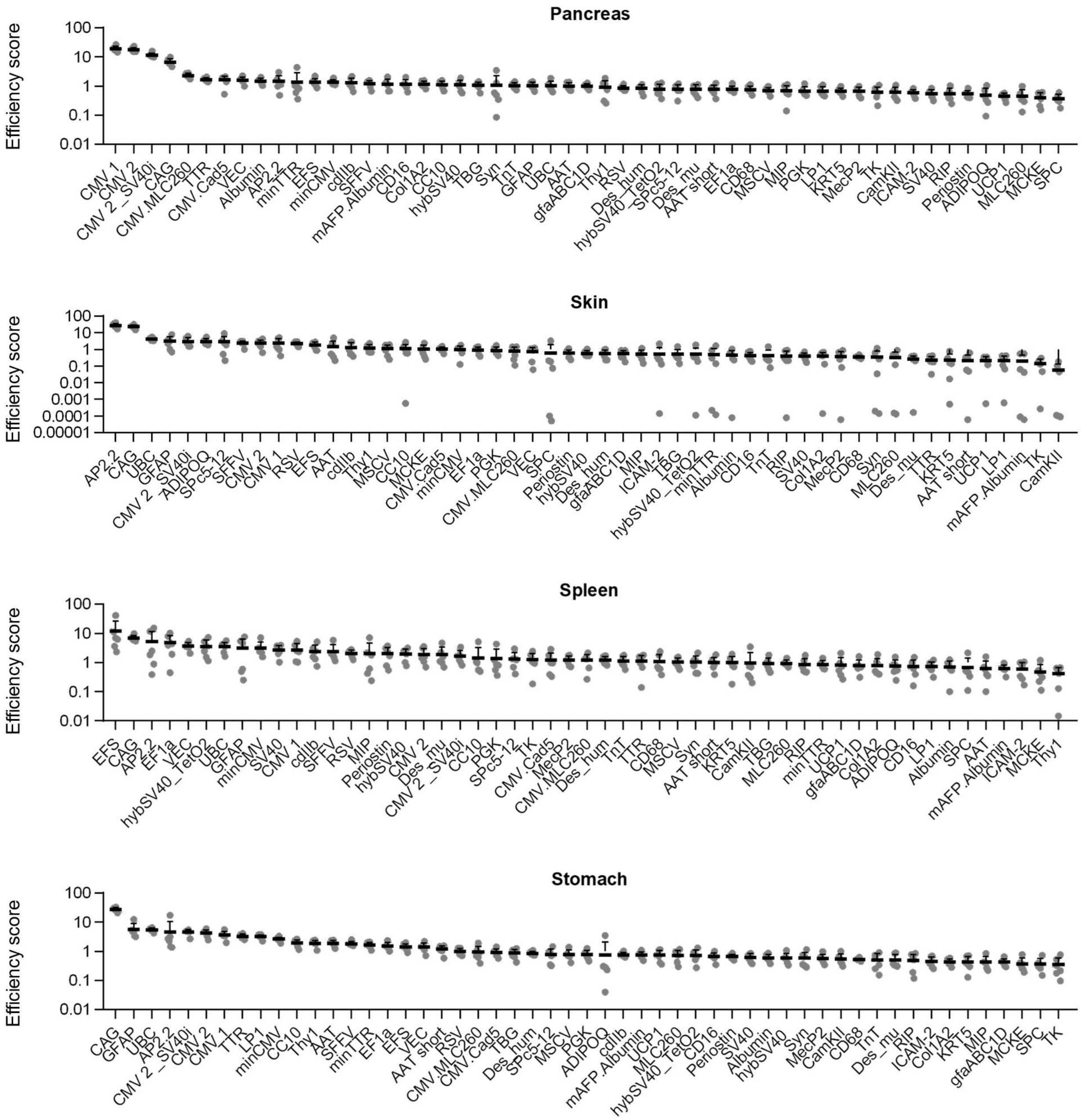


**Figure S5. Ranked efficiency scores of 53 promoters across all 16 tissues examined.** For each tissue sample, efficiency scores were calculated and ranked according to the average score over six replicate animals. Efficiency scores are shown for the following tissues: pancreas, skin, spleen and stomach. Replicate values (N=6) are plotted with mean and standard deviation.

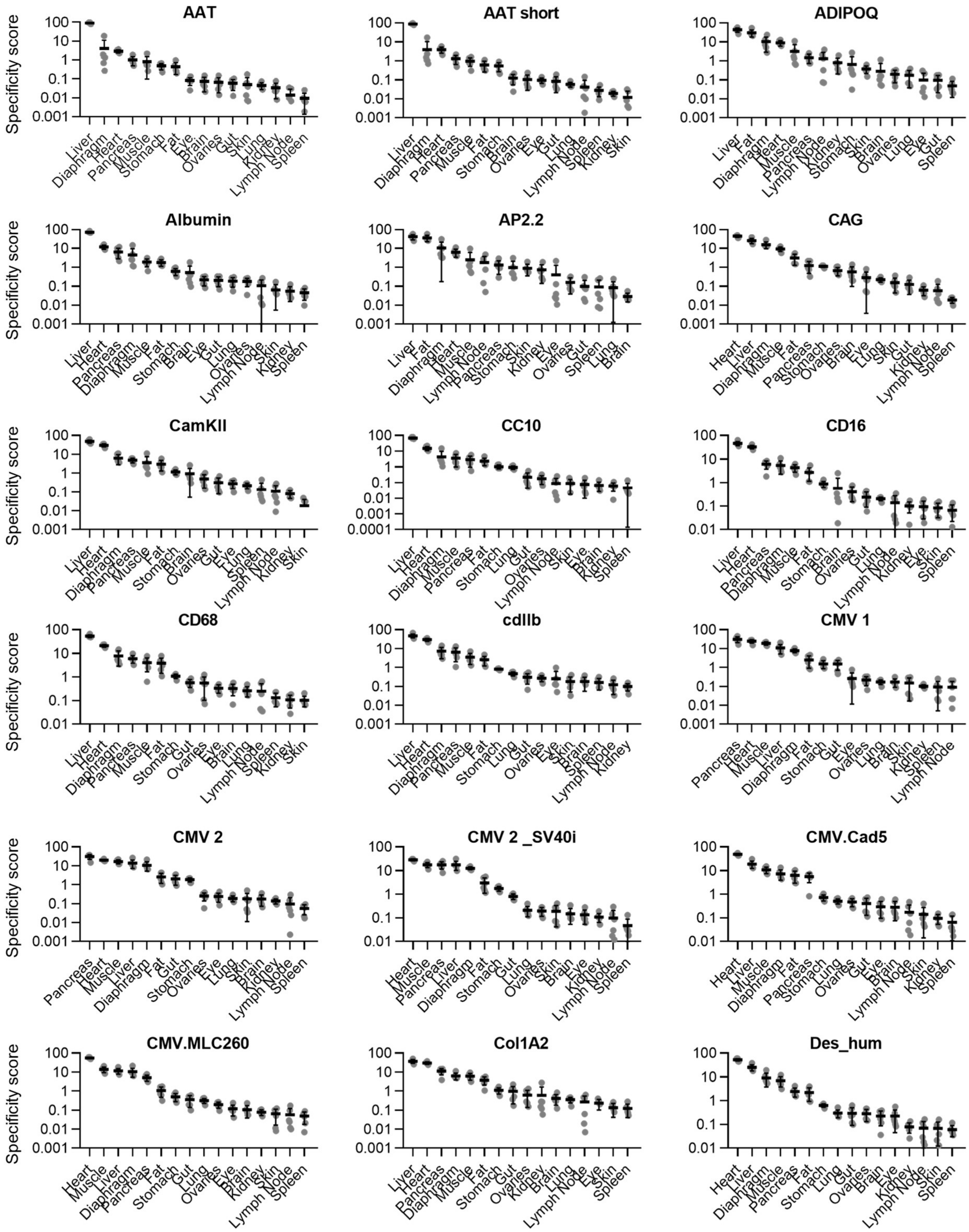


**Figure S6. Specificity scores calculated for all 53 promoters included in the promoter library.** Specificity scores were calculated for all 16 tissues for each individual promoter and

ranked by the mean score across six replicate animals. Replicate scores (N=6) are plotted with mean and standard deviation. Continued in Figures S7 and S8.

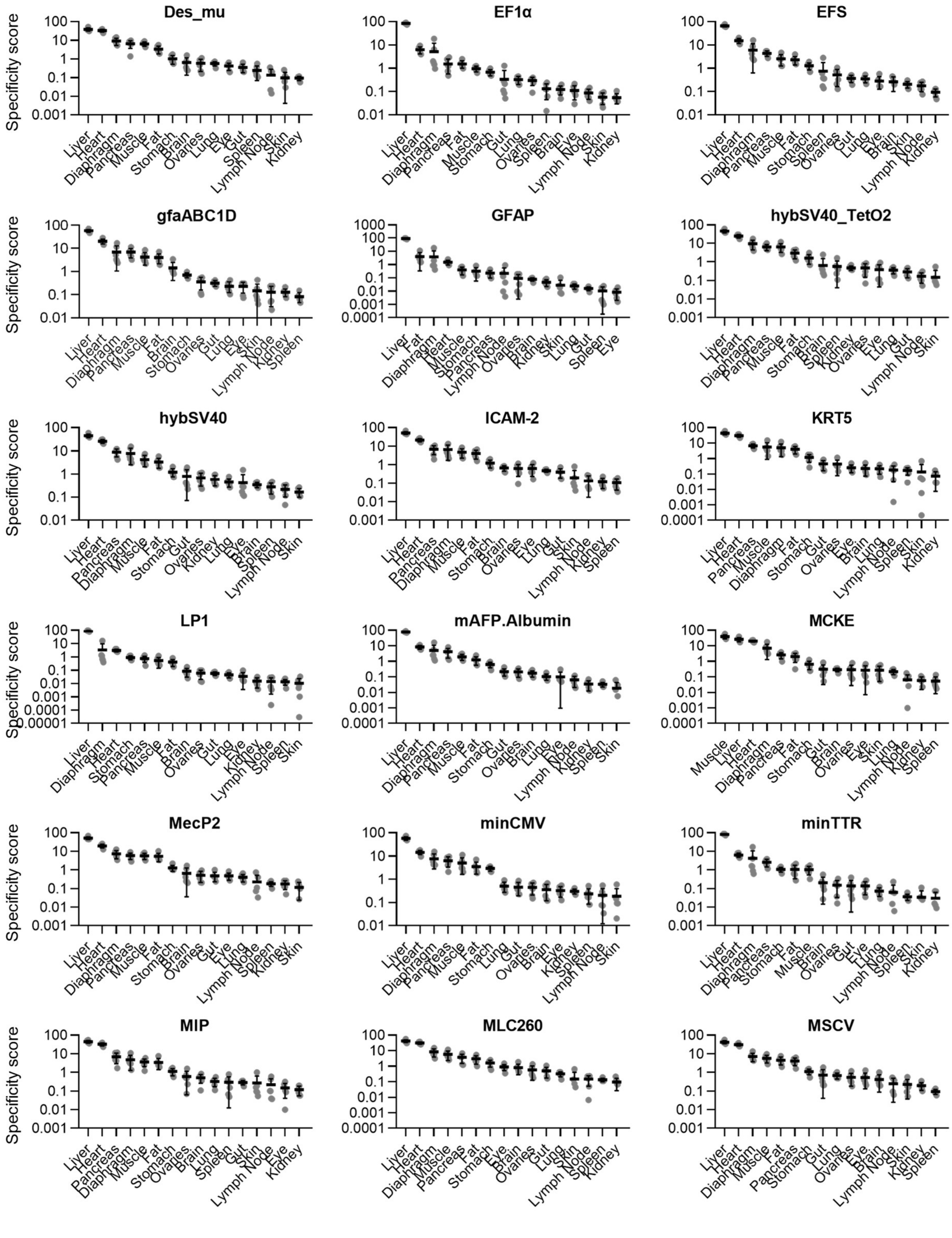


**Figure S7 (continued from S6). Specificity scores calculated for all 53 promoters included in the promoter library.**

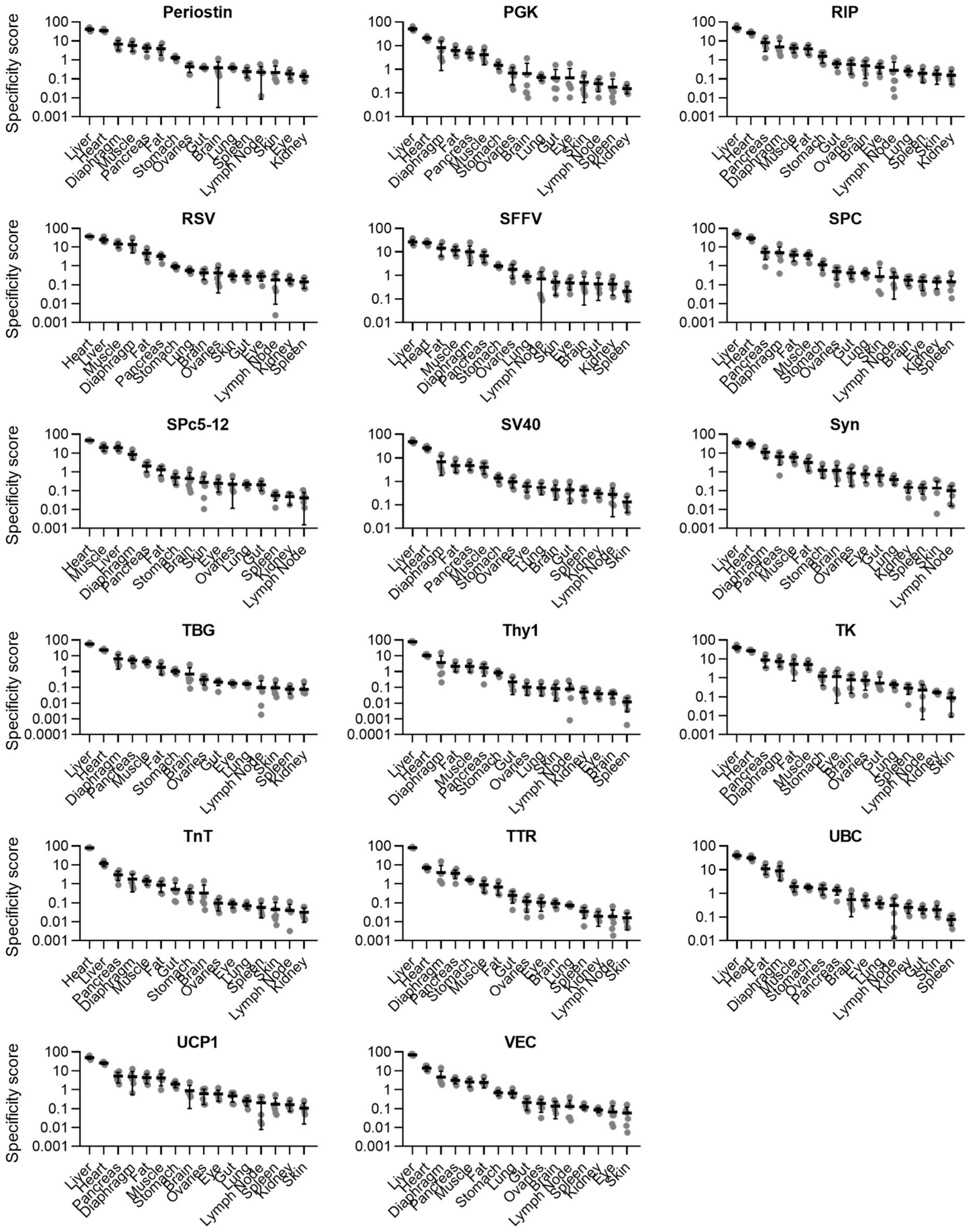


**Figure S8 (continued from S6 and S7). Specificity scores calculated for all 53 promoters included in the promoter library.**

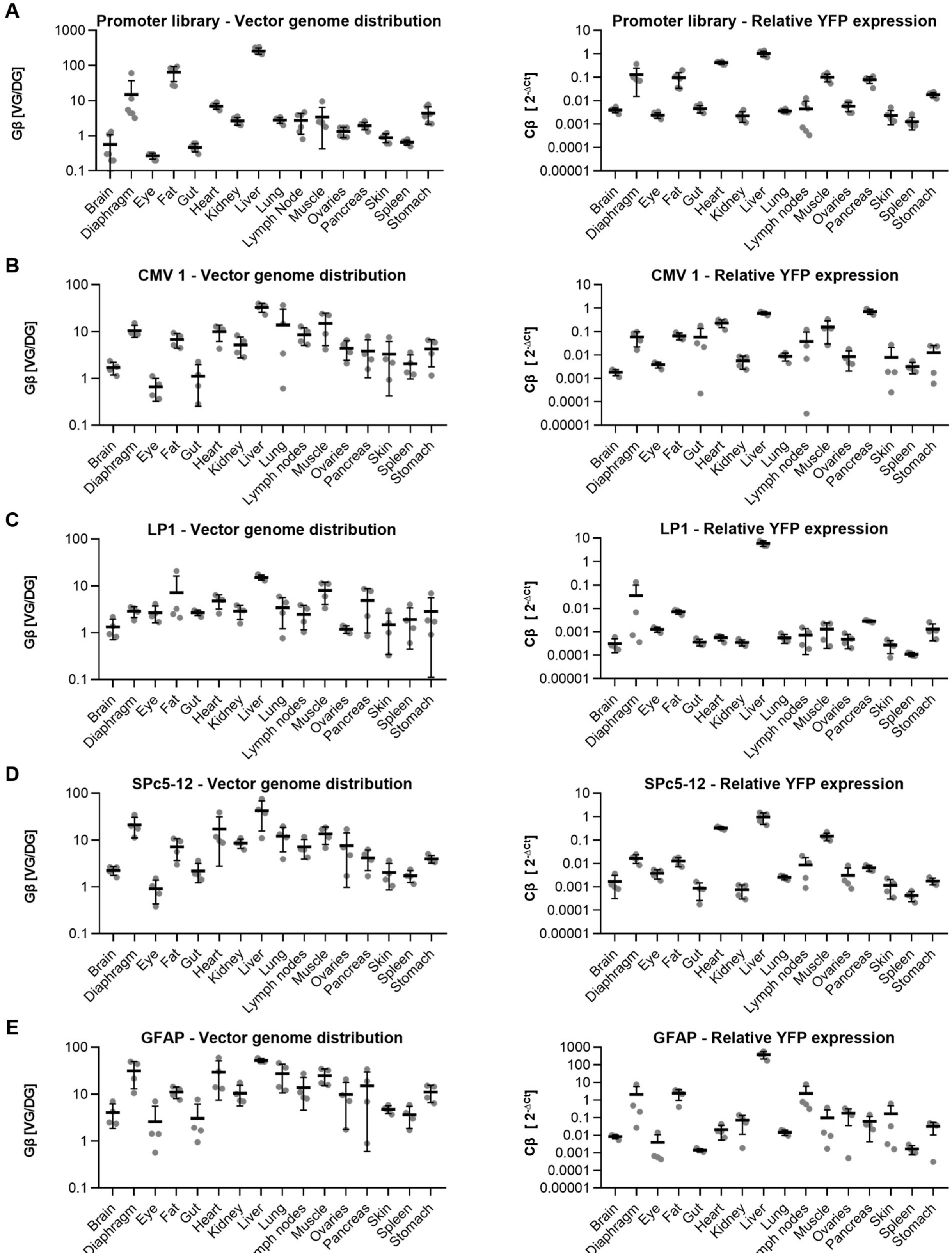


**Figure S9. Vector genome biodistribution and relative *eyfp* expression measured for the promoter library and validation screens.** Distribution of vector genomes *Gβ* [VG/DG] and

relative *eyfp* expression $C\beta$ [$2^{-\Delta Ct}$] measured across all 16 tissues examined for (A) the promoter library (N=6 replicate animals), or the individual constructs with the promoters (B) CMV1 (N=4), (C) LP1 (N=4), (D) SPc5-12 (N=4), or (E) GFAP (N=4).

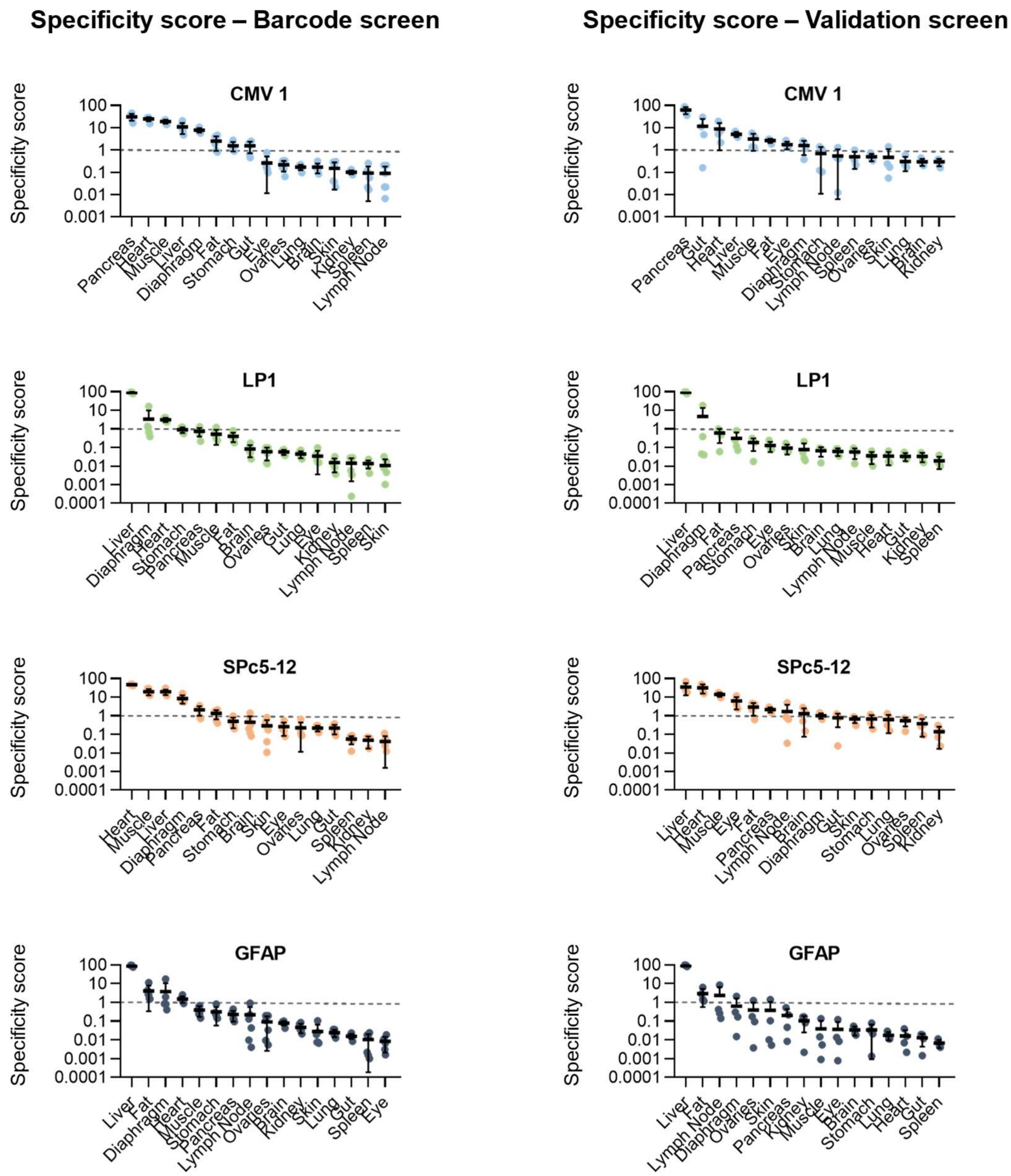


**Figure S10. Specificity scores calculated for CMV 1, LP1, SPc5-12 and GFAP from the library barcode and the single-construct validation screen.**

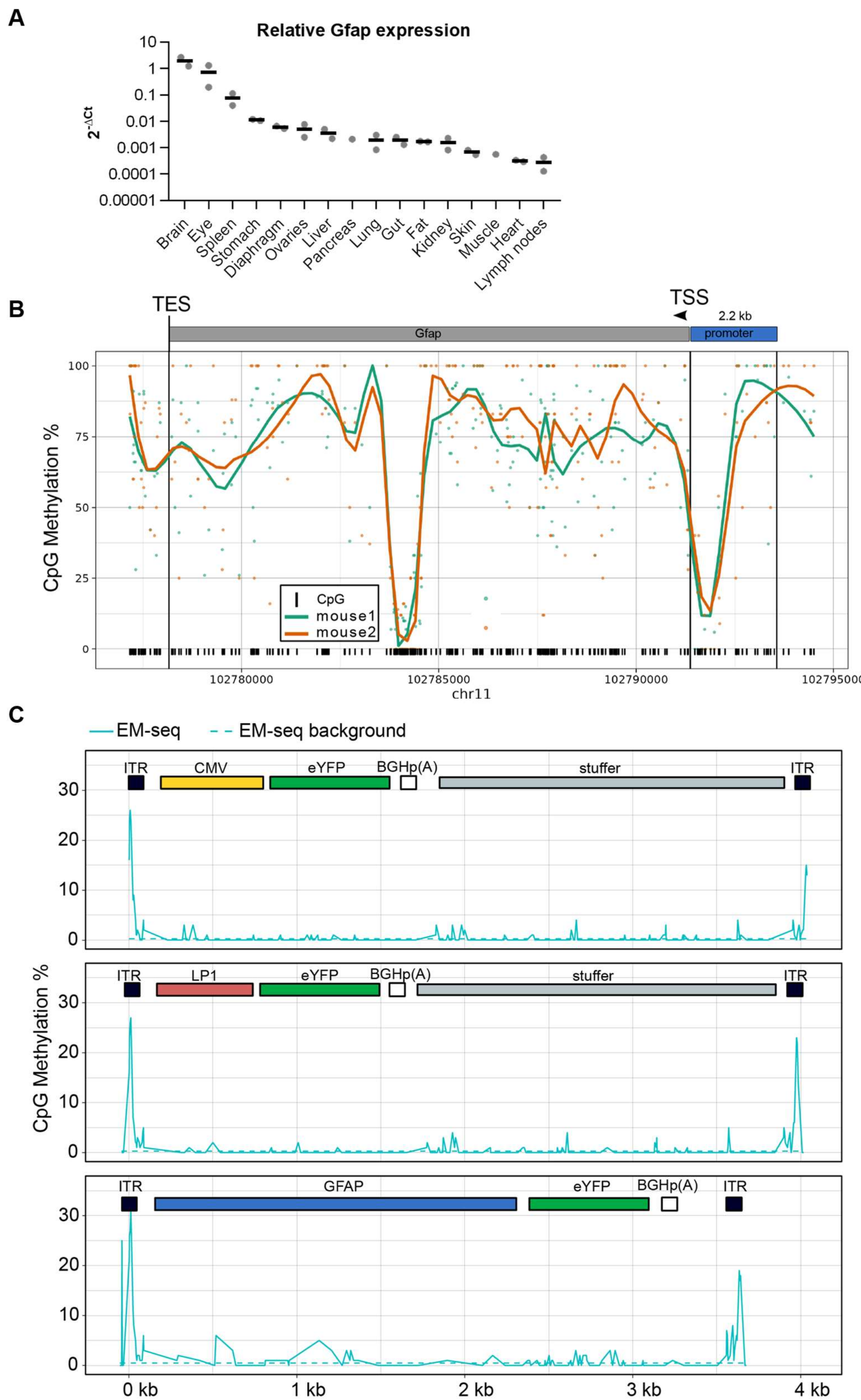


**Figure S11. Evaluation of endogenous Gfap expression and methylation status of endogenous or AAV-delivered promoters.** (A) Relative expression of the endogenous *Gfap*

normalized to *PolR2A* housekeeper expression. Data from n=2 untreated mice. (B) Liver CpG methylation frequency along the endogenous mouse *Gfap* gene on chromosome 11 of GRCm39 (NC_000077.7:102778162-102791368) as determined from bisulfite sequencing data derived from publicly available data (SRR24770973, SRR24770974) (n=2 untreated mice). The mouse *Gfap* promoter is here defined as the 2.2 kb sequence upstream of the transcription start site (TSS; NC_000077.7:102791368-102793568). Every dot represents the methylation frequency of a single CpG site. The line represents a loss of the individual CpG sites. TES, transcription end site. (C) Methylation frequency along the AAV vector genomes in the liver containing a CMV, LP1, or GFAP promoter as determined 14 days post-delivery through EM-seq. The dotted line represents the mean methylation frequency determined from unmethylated DNA.

A

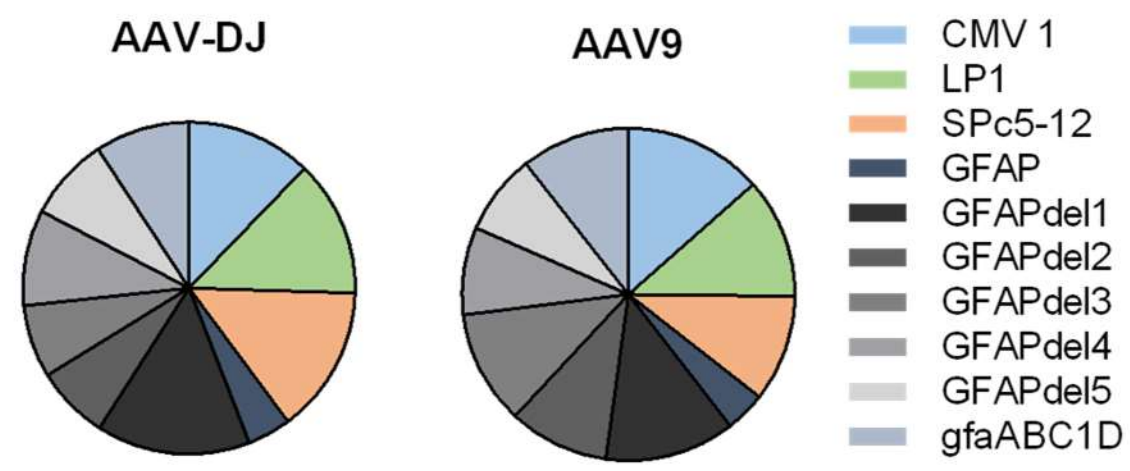


B

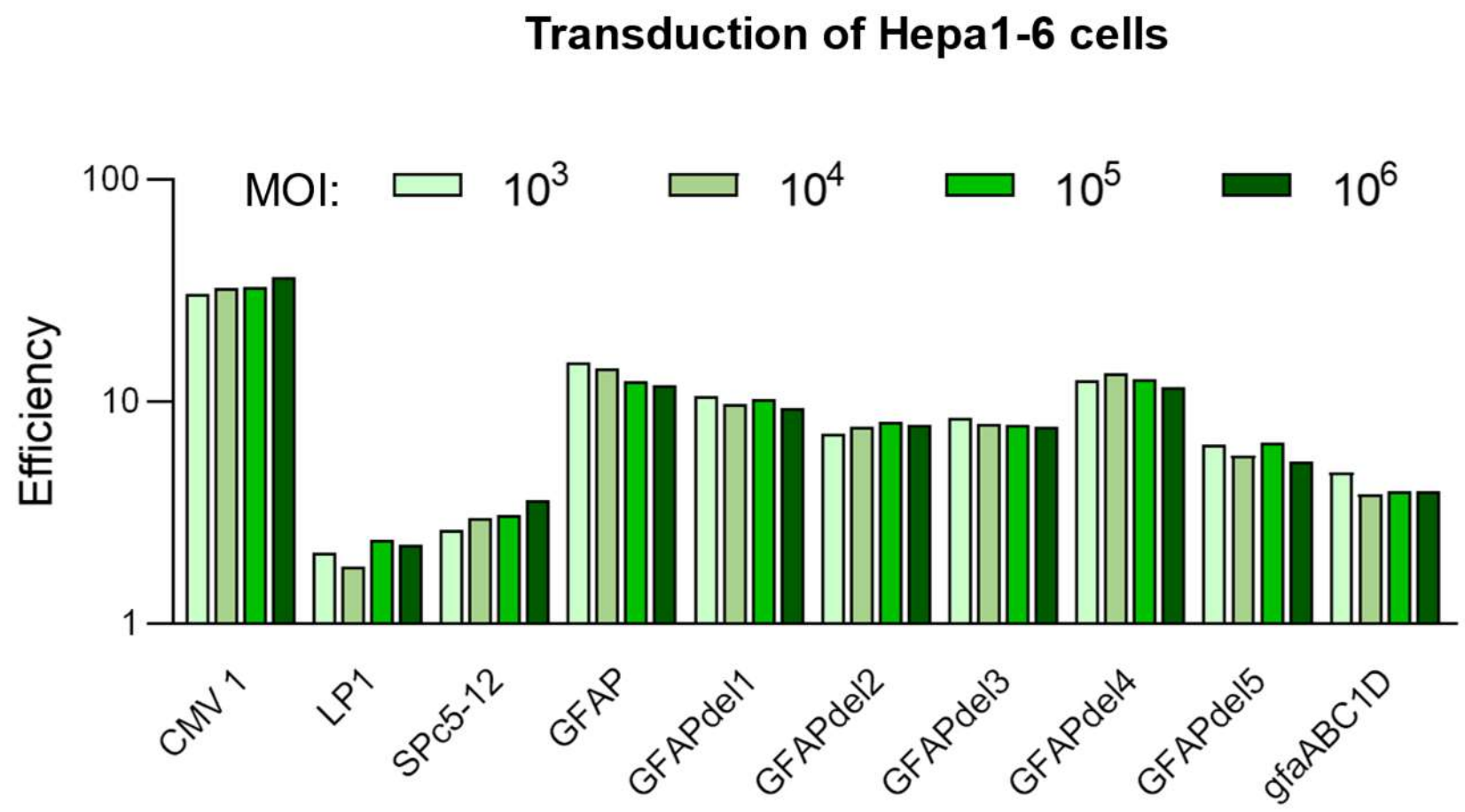


**Figure S12. Promoter libraries for the study of GFAP promoter deletion variants.** (A) NGS-based characterization of the composition of the AAV-DJ- and AAV9-based promoter mini-libraries. (B) Relative promoter efficiency as determined by barcode sequencing after transduction of Hepa1-6 cells. Hepa1-6 cells were transduced with the AAV-DJ mini-library using different MOIs ($10^3$, $10^4$, $10^5$ and $10^6$). DNA and RNA were extracted on day 3 post-transduction, followed by barcode readout on DNA and cDNA (RNA) levels. Efficiency scores were calculated as before.

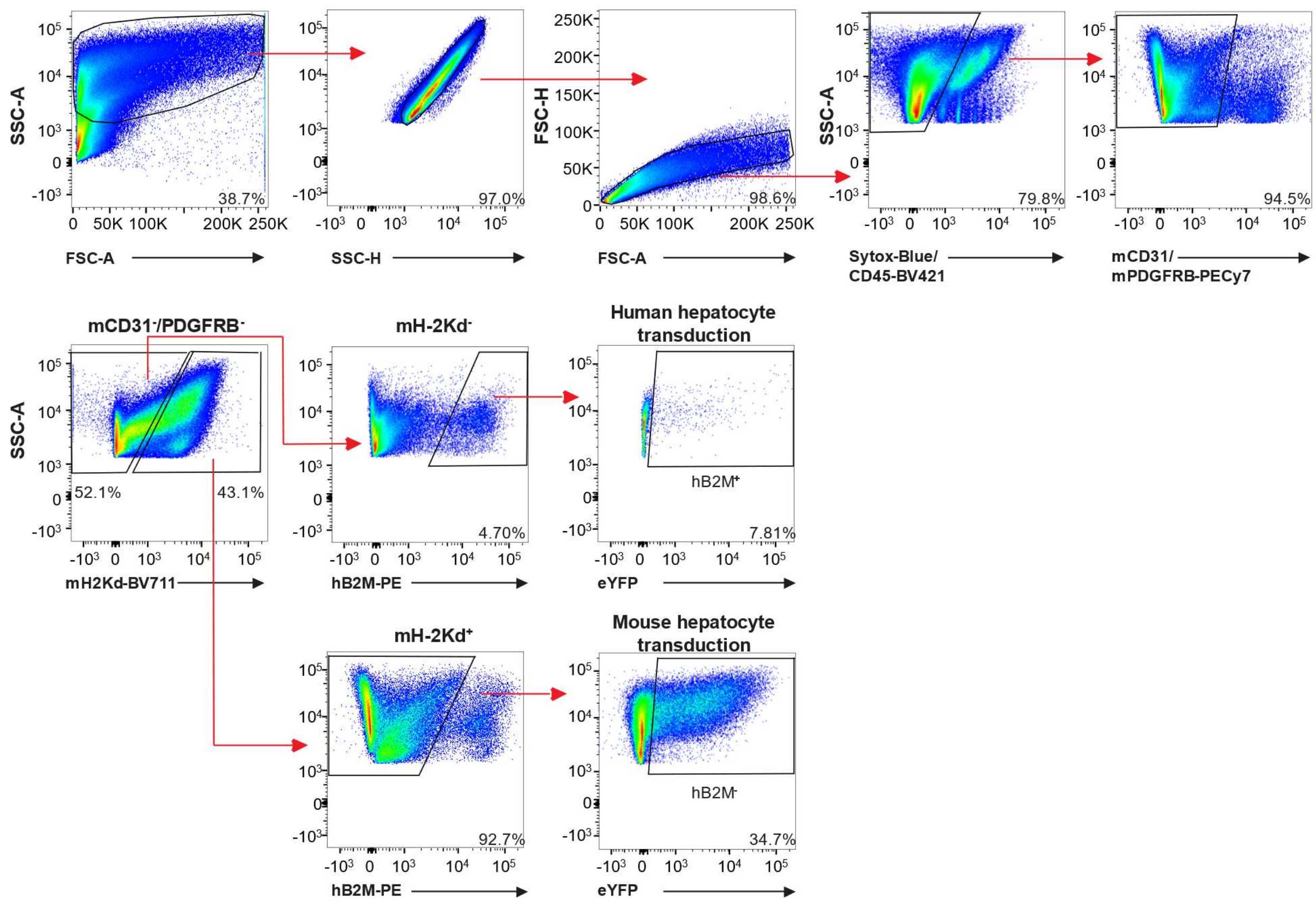


**Figure S13. FACS-based isolation of human and murine hepatocytes from FRGN mouse transduced with the AAV9 promoter mini-library.** Flow cytometry analysis of human and mouse hepatocytes released from a humanized FRGN mouse two weeks after injection with the AAV9 promoter mini-library. Non-parenchymal cells were excluded using CD45-BV421 and mCD31/mPDGFRB-PECy7 markers. Human hepatocytes were identified and collected from the mH2Kd-BV711-negative, hB2M-PE-positive gate, while mouse hepatocytes were collected from the mH2Kd-BV711-positive, hB2M-PE-negative gate.
