# Supplementary material for: Identification of a robust promoter in mouse and human hepatocytes by in vivo biopanning of a barcoded AAV library
Source: Mol Ther. Author manuscript; Available in PMC 2026 Apr 21. (PMC12443165; doi:10.1016/j.ymthe.2025.04.027)
Supplement: Figures S1-S13 [file NIHMS2108476-supplement-Figures_S1-S13.pdf]

YMTHE, Volume *33*

Supplemental Information

Identification of a robust promoter in mouse and human hepatocytes by *in vivo* biopanning of a barcoded AAV library

Jonas Becker, Claire Domenger, Pervinder Choksi, Chiara Krämer, Conradin Baumgartl, Olena Maiakovska, Jae-Jun Kim, Jonas Weinmann, Georg Huber, Florian Schmidt, Christian Thirion, Oliver J. Müller, Holger Willenbring, and Dirk Grimm

Table S1: Detailed information on all 53 promoters included in the primary promoter library. Information on promoter name, description, supposed tissue specificity, sequence, length, origin and employed stuffer size is provided.

Table S2: Putative transcription factor binding sites detected within the GFAP promoter. (Sheet 1) FIMO output for transcription factor MEMEs using the JASPAR Core dataset for all deposited TFs from human and mouse origin[109]. (Sheet 2) Subsection of Sheet 1 with matching transcription factors in the Zhou *et al*. tissue-specificity scores for liver[110].

Table S3: Parameters used for barcode extraction and NGS analysis of the promoter library dataset. For more details, see Rapti *et al*. [42].

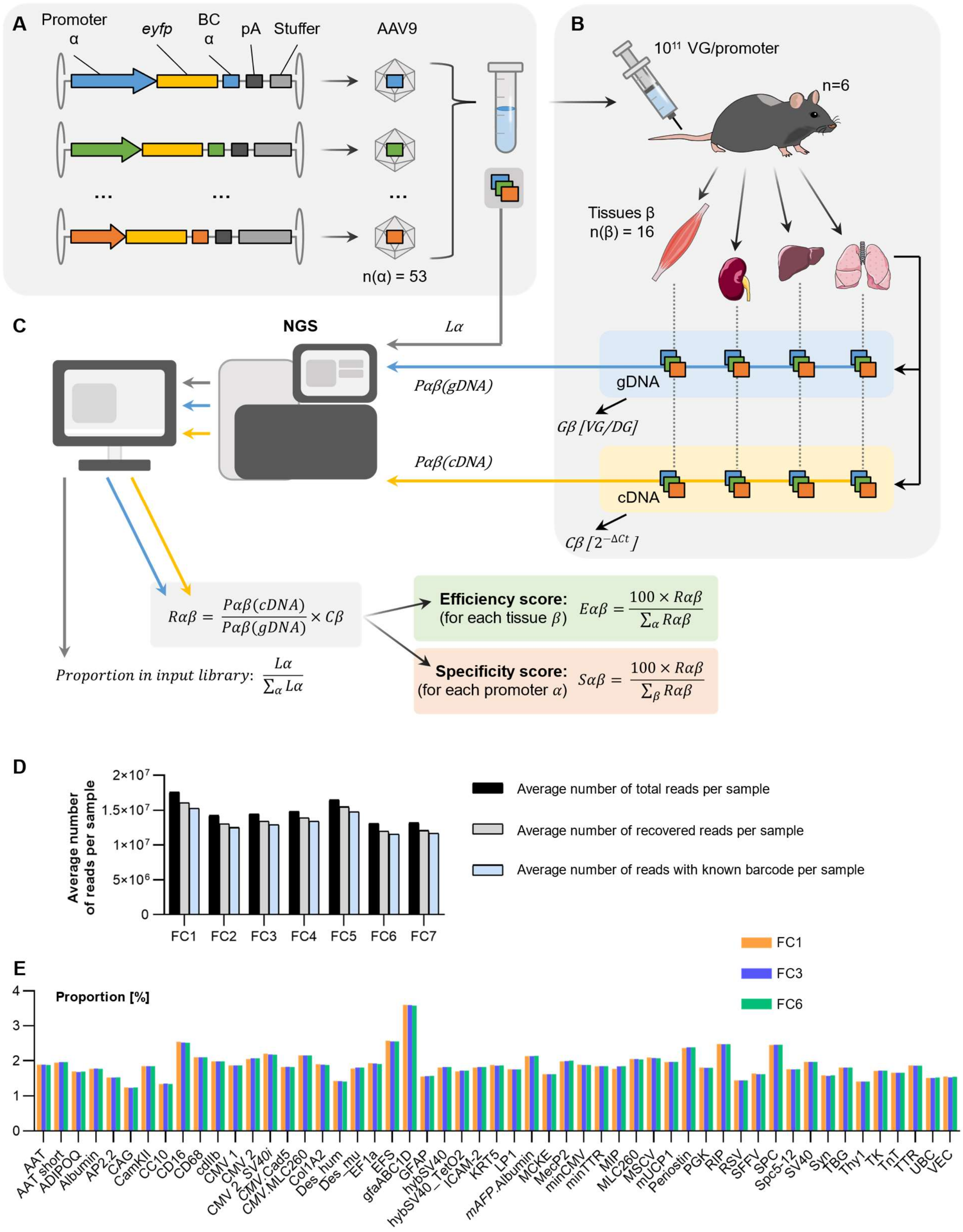

Figure S1. Detailed normalization strategy and read proportions for the barcoded promoter library screen. (A-B) A library of 53 different promoters (α_1-α_{53}) was cloned, produced and

screened in mice as described in Figure 1. Two weeks after injection, 16 different tissues (β_1-β_{16}) were harvested and subjected to DNA and RNA extraction. For each tissue *β*, the vector genome copy number per cell was measured by ddPCR from gDNA (*Gβ*), and relative bulk *eyfp* expression was measured by qPCR (*Cβ*). (C) Illumina sequencing was performed to evaluate the contribution of individual promoters *α* to bulk *eyfp* expression. Therefore, barcode-containing amplicons were generated and sequenced to obtain (i) *Lα,* the relative barcode proportions within the input vector library, (ii) *Pαβ(gDNA)*, the barcode proportions in gDNA samples extracted from each tissue *β*, which gives the proportions of individual promoters *α* in tissue *β* at the gDNA level, and (iii) *Pαβ(cDNA)*, the barcode proportions in cDNA samples derived from each tissue *β*, which gives the proportions of individual promoters *α* from each tissue *β* at the cDNA level. To calculate *Rαβ*, the relative normalized expression of each promoter *α* within each organ *β*, *i.e.*, *Pαβ(cDNA),* was divided by *Pαβ(gDNA)* and subsequently multiplied by the relative bulk *eyfp Cβ* of that tissue sample. Finally, *Rαβ* was used to calculate efficiency and specificity scores. For efficiency scores, *Rαβ* values within each tissue sample were normalized to the sum of *Rαβ* values of all 53 promoters within that tissue, followed by scaling by 100 to yield values from 0-100. For specificity scores, *Rαβ* values of a promoter α were normalized to the sum of *Rαβ* across all 16 tissue samples within each replicate animal, also followed by scaling by 100 to yield values from 0-100. (D) Total recovered and correctly assigned read numbers obtained from Illumina sequencing performed in seven flow cells (FC1-7). (E) Barcode proportions of the injected promoter library (Lα) were sequenced three times, with the three samples distributed among flow cells (FC) 1, 3 and 6. Mean values of each barcode fraction were calculated, yielding *Lα*.

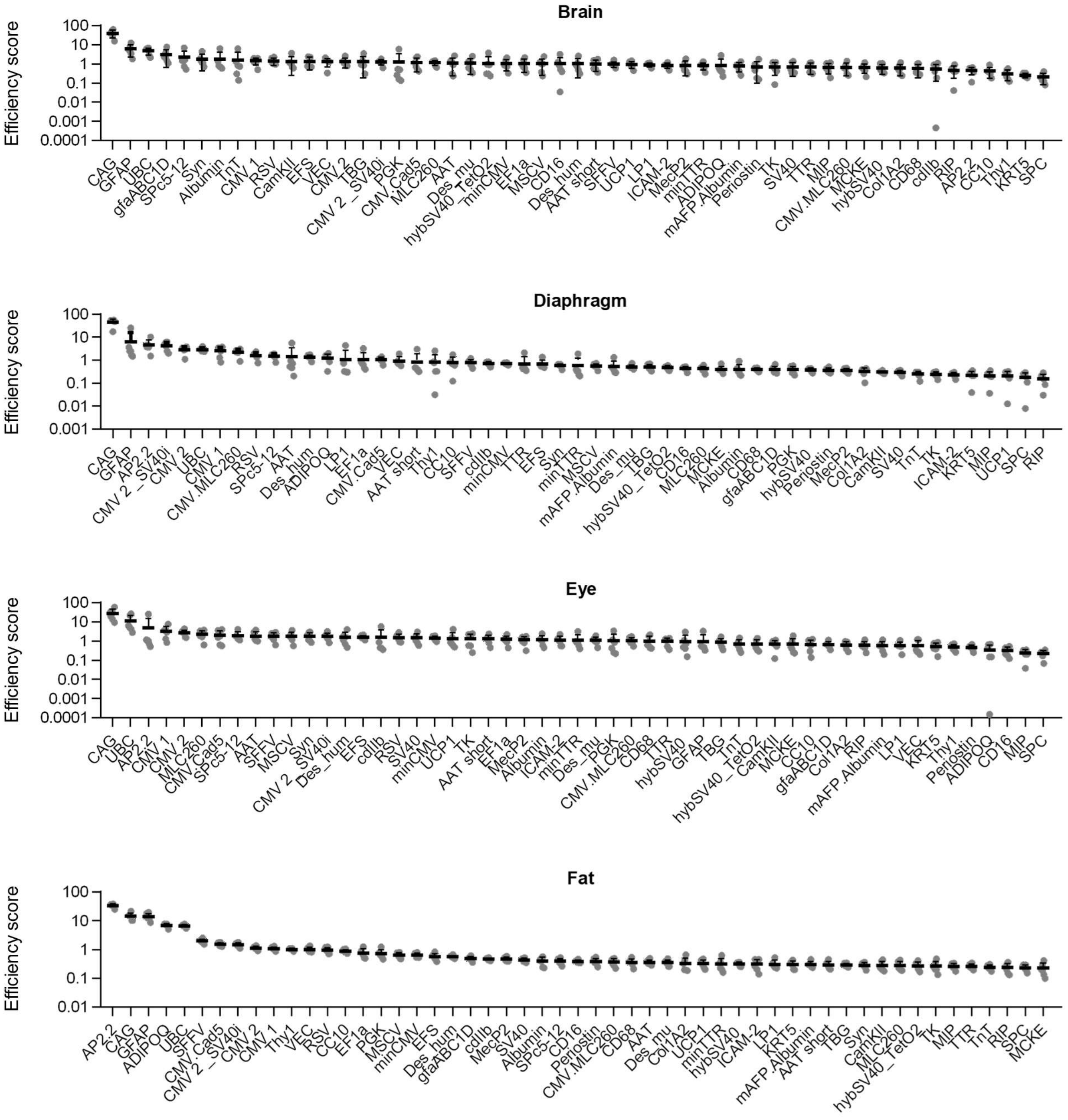

Figure S2. Ranked efficiency scores of 53 promoters across all 16 tissues examined. For each tissue sample, efficiency scores were calculated and ranked according to the average score over six replicate animals. Efficiency scores are shown for the following tissues: brain, diaphragm, eye and (white) fat tissue. Replicate values (N=6) are plotted with mean and standard deviation.

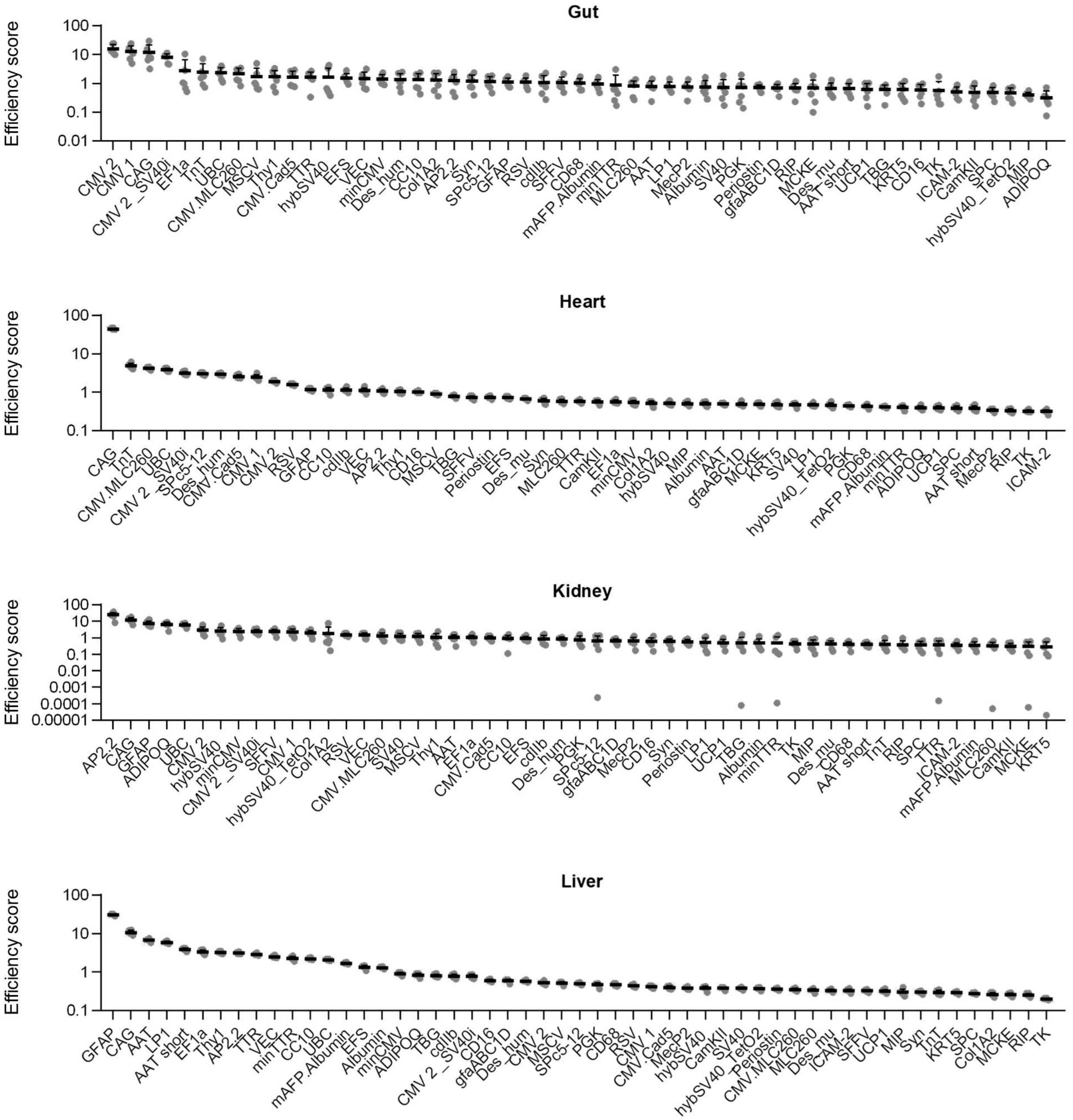

Figure S3. Ranked efficiency scores of 53 promoters across all 16 tissues examined. For each tissue sample, efficiency scores were calculated and ranked according to the average score over six replicate animals. Efficiency scores are shown for the following tissues: gut (duodenum), heart, kidney and liver samples. Replicate values (N=6) are plotted with mean and standard deviation.

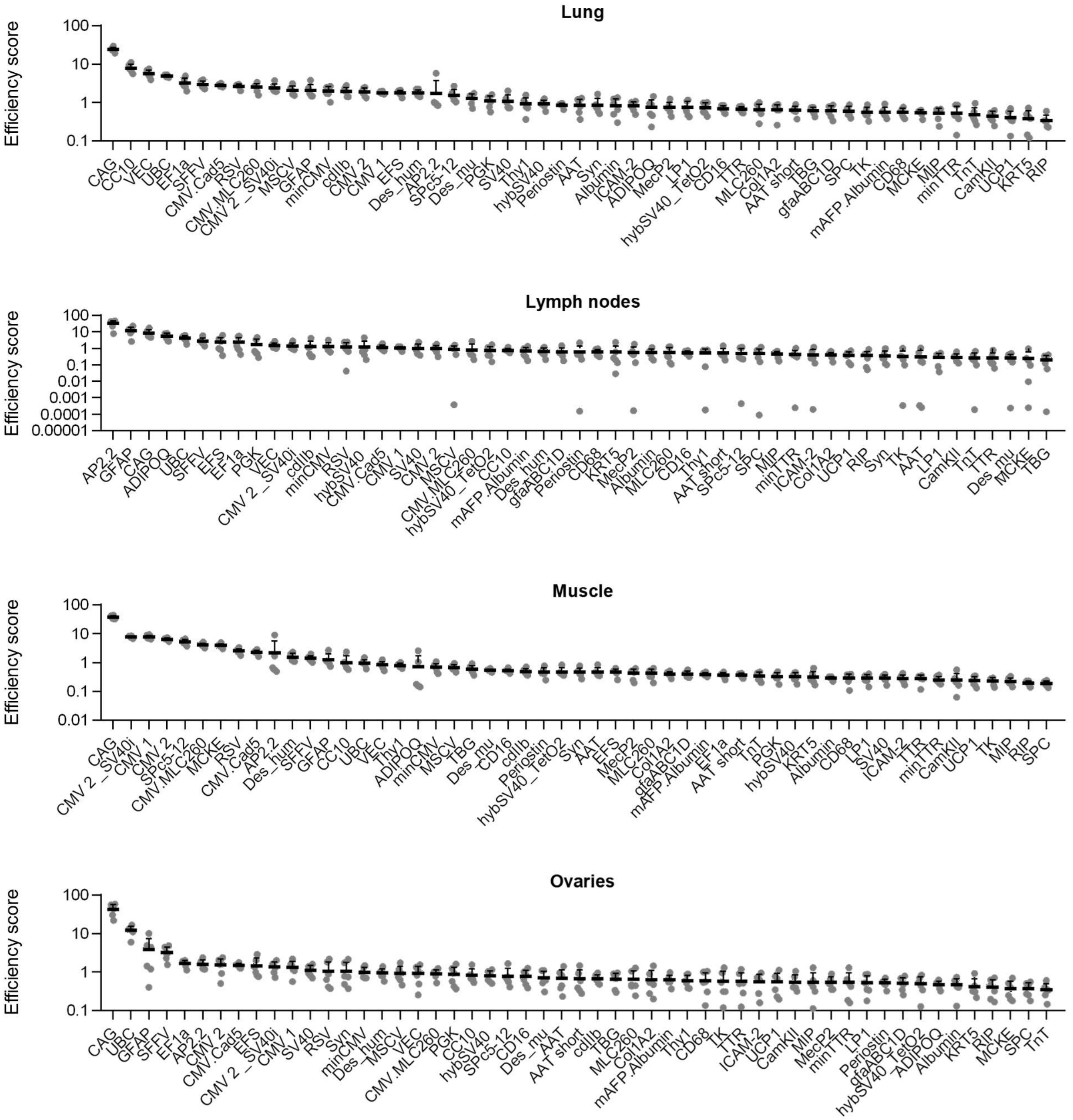

Figure S4. Ranked efficiency scores of 53 promoters across all 16 tissues examined. For each tissue sample, efficiency scores were calculated and ranked according to the average score over six replicate animals. Efficiency scores are shown for the following tissues: lung, lymph nodes (inguinal), (skeletal) muscle (*quadriceps femoris*) and ovaries. Replicate values (N=6) are plotted with mean and standard deviation.

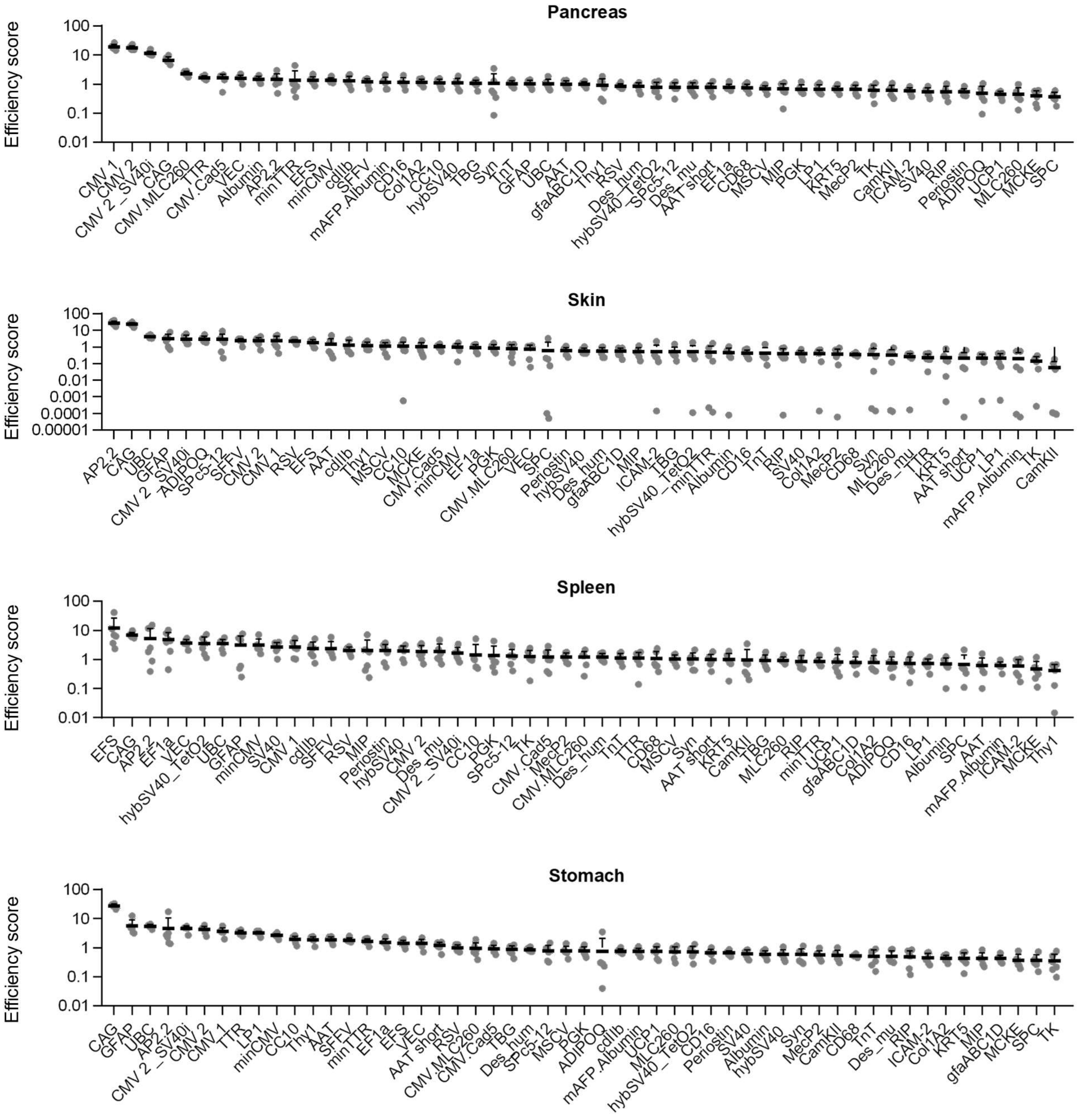

Figure S5. Ranked efficiency scores of 53 promoters across all 16 tissues examined. For each tissue sample, efficiency scores were calculated and ranked according to the average score over six replicate animals. Efficiency scores are shown for the following tissues: pancreas, skin, spleen and stomach. Replicate values (N=6) are plotted with mean and standard deviation.

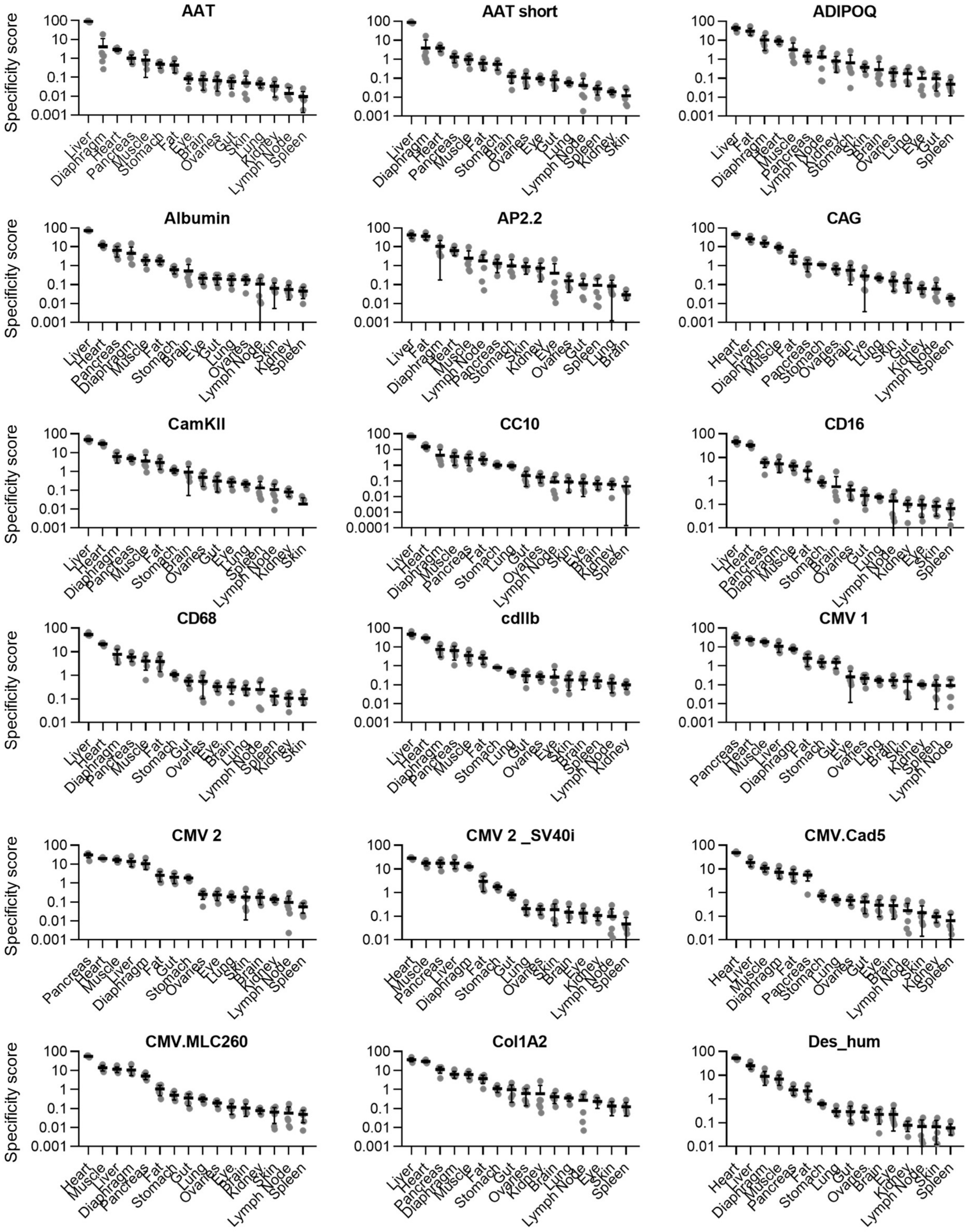

Figure S6. Specificity scores calculated for all 53 promoters included in the promoter library. Specificity scores were calculated for all 16 tissues for each individual promoter and

ranked by the mean score across six replicate animals. Replicate scores (N=6) are plotted with mean and standard deviation. Continued in Figures S7 and S8.

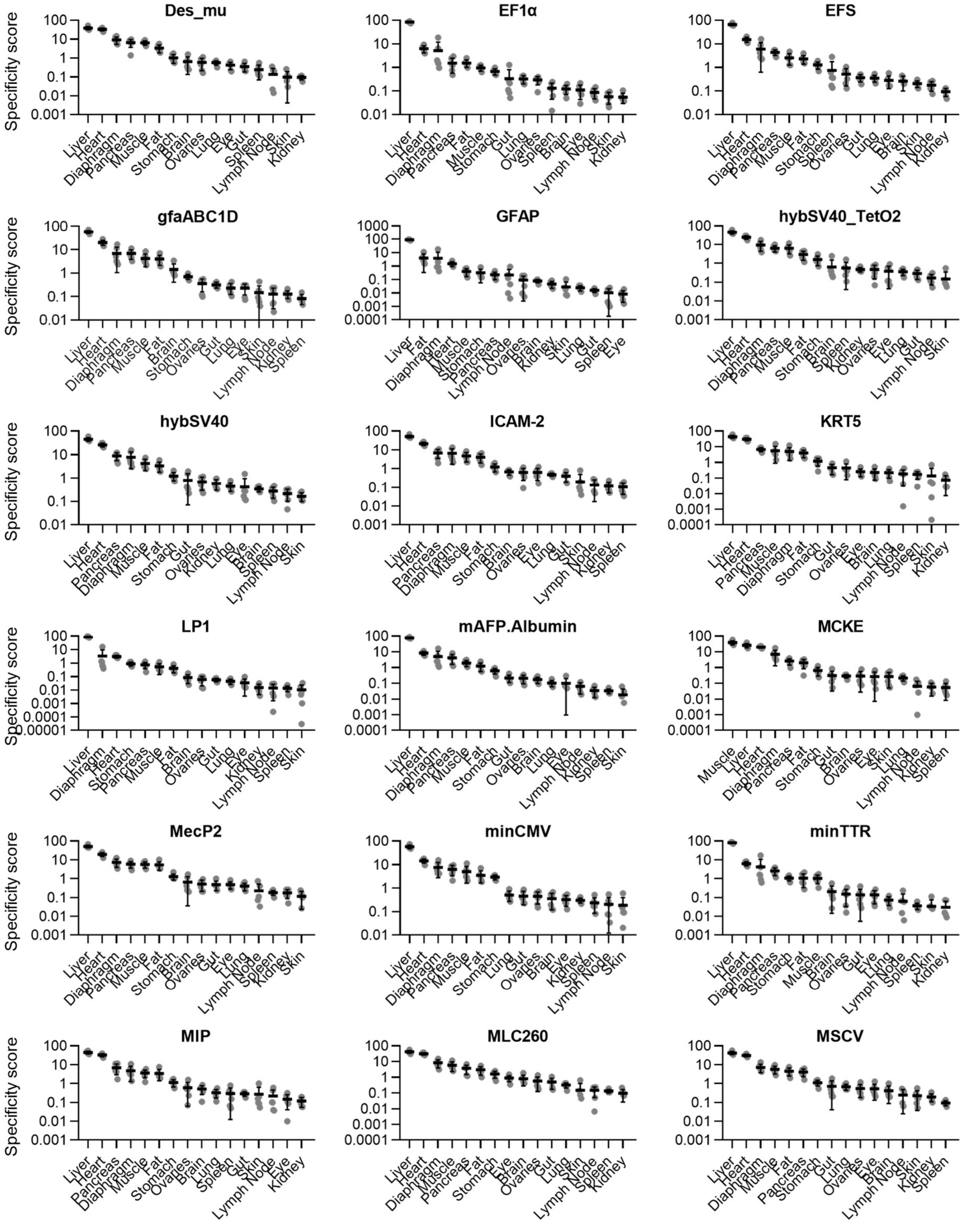

Figure S7 (continued from S6). Specificity scores calculated for all 53 promoters included in the promoter library.

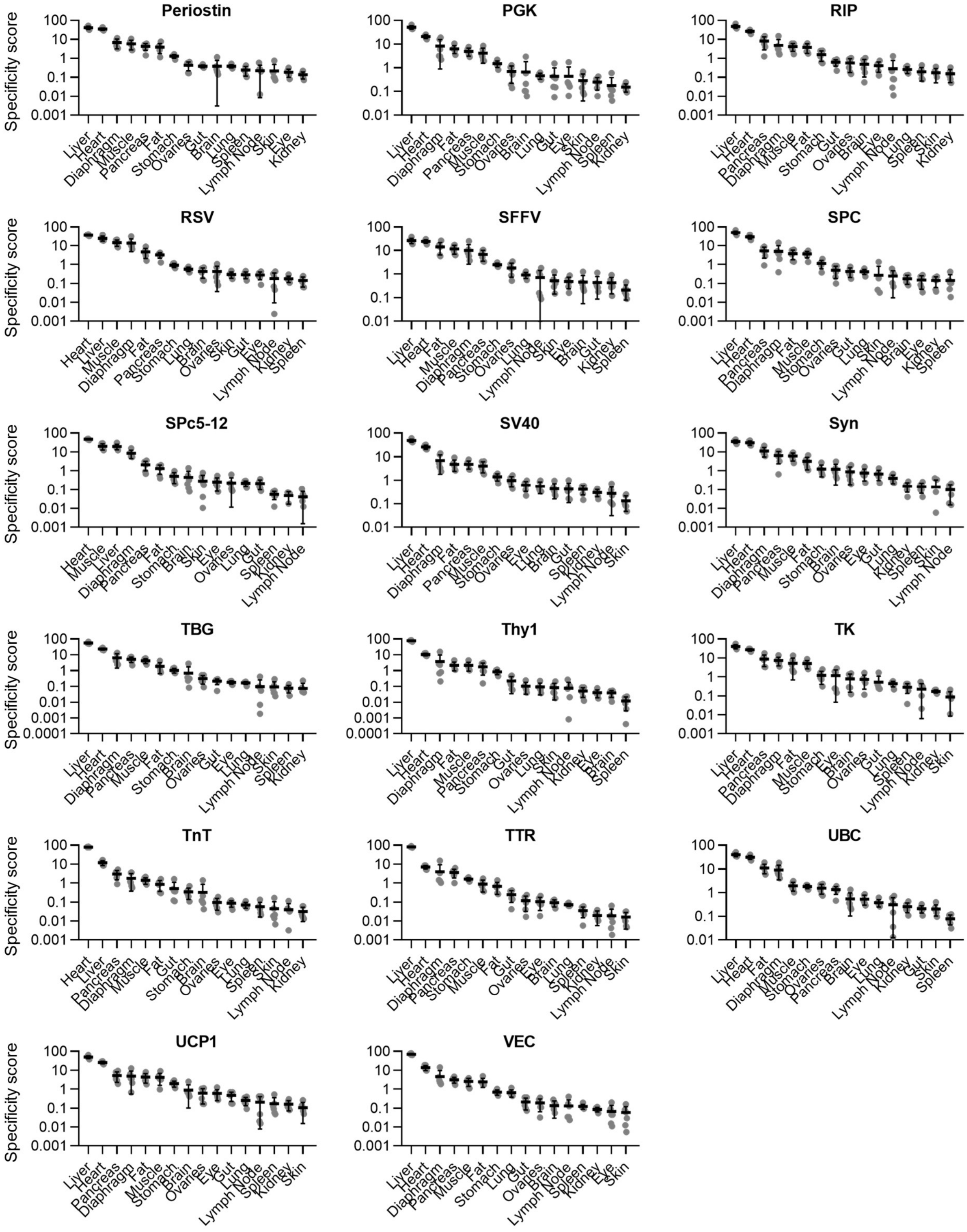

Figure S8 (continued from S6 and S7). Specificity scores calculated for all 53 promoters included in the promoter library.

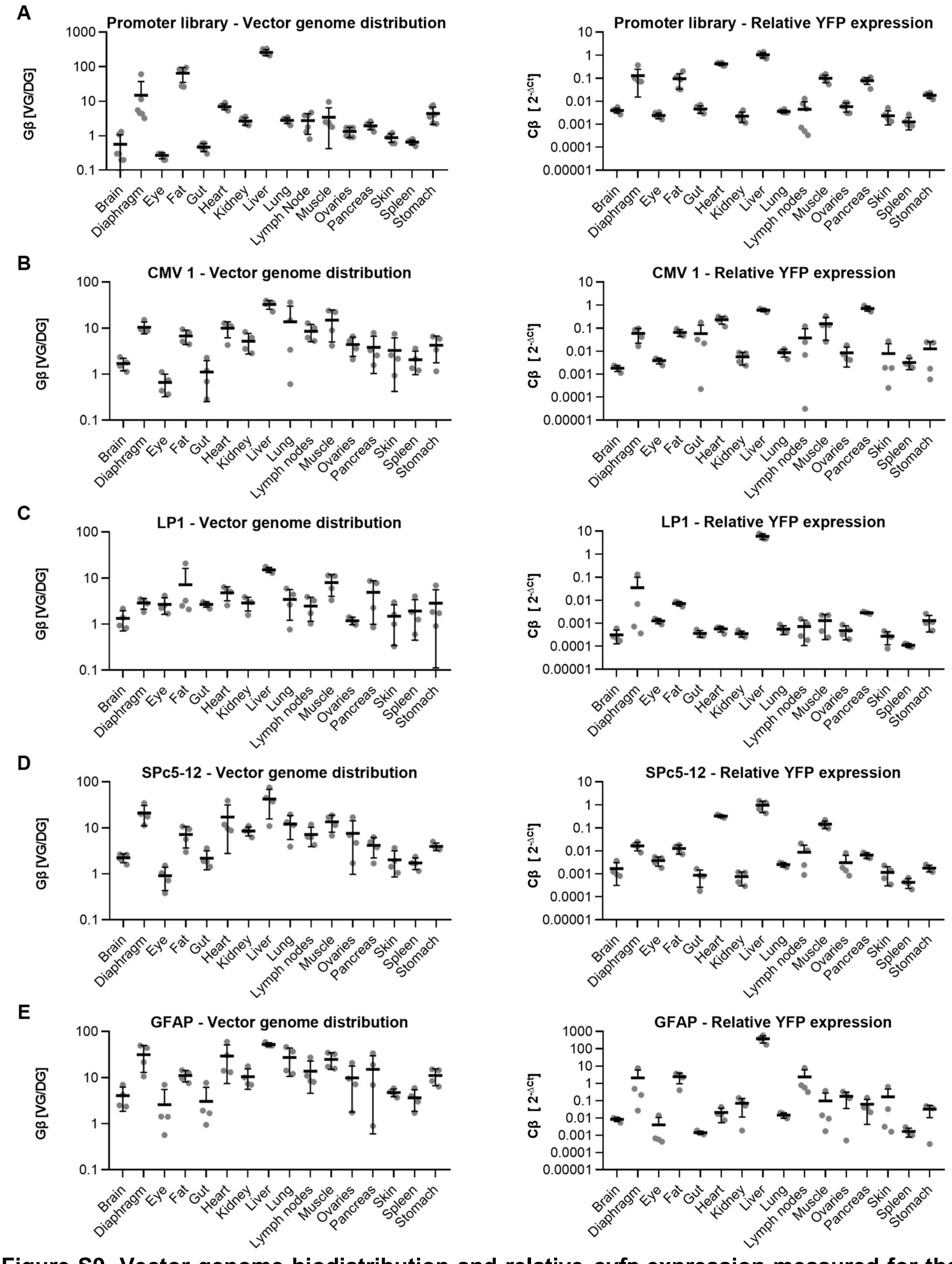

Figure S9. Vector genome biodistribution and relative *eyfp* expression measured for the promoter library and validation screens. Distribution of vector genomes *Gβ* [VG/DG] and

relative *eyfp* expression *Cβ* [$2^{-\Delta Ct}$] measured across all 16 tissues examined for (A) the promoter library (N=6 replicate animals), or the individual constructs with the promoters (B) CMV1 (N=4), (C) LP1 (N=4), (D) SPc5-12 (N=4), or (E) GFAP (N=4).

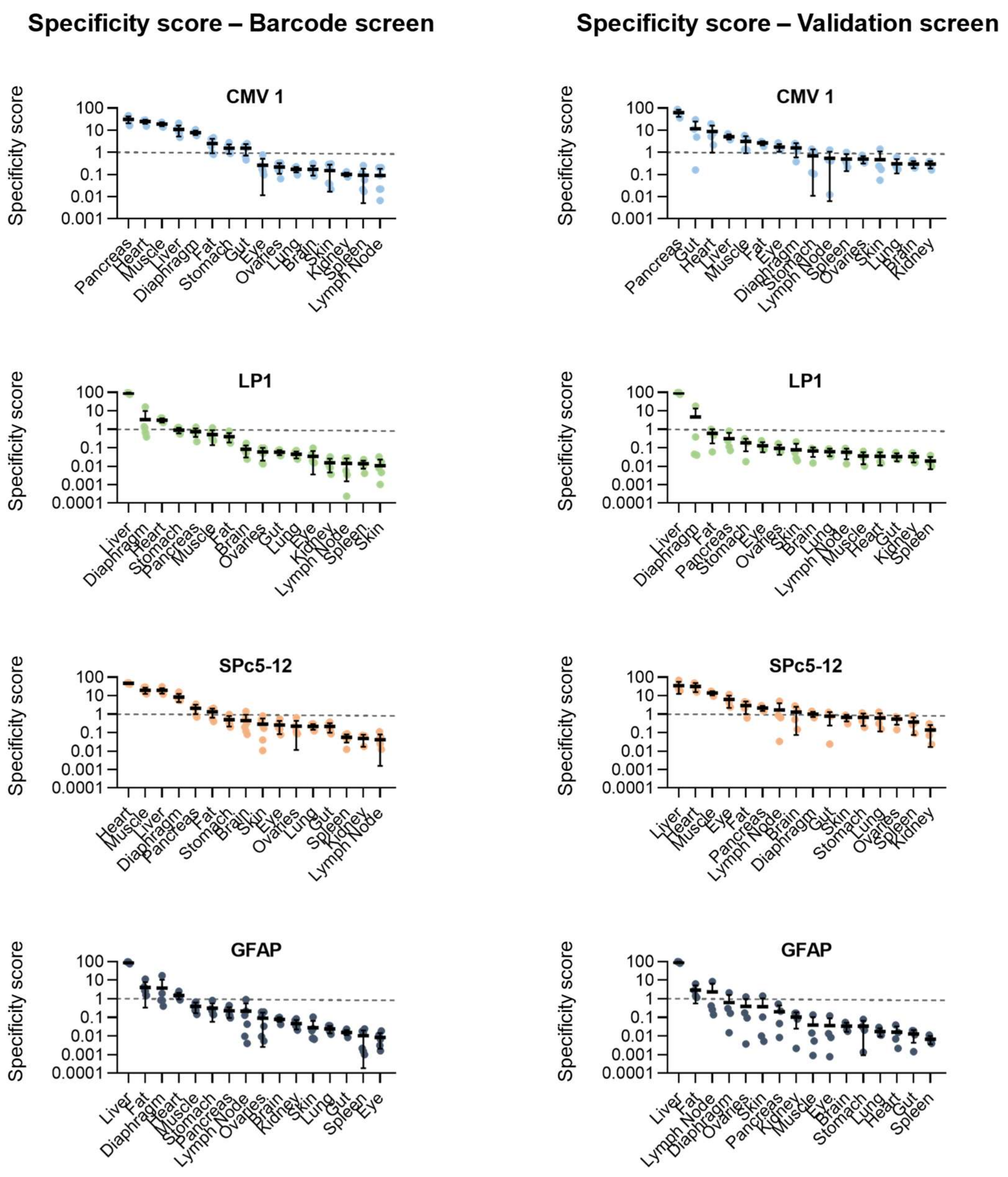

Figure S10. Specificity scores calculated for CMV 1, LP1, SPc5-12 and GFAP from the library barcode and the single-construct validation screen.

A

B

C

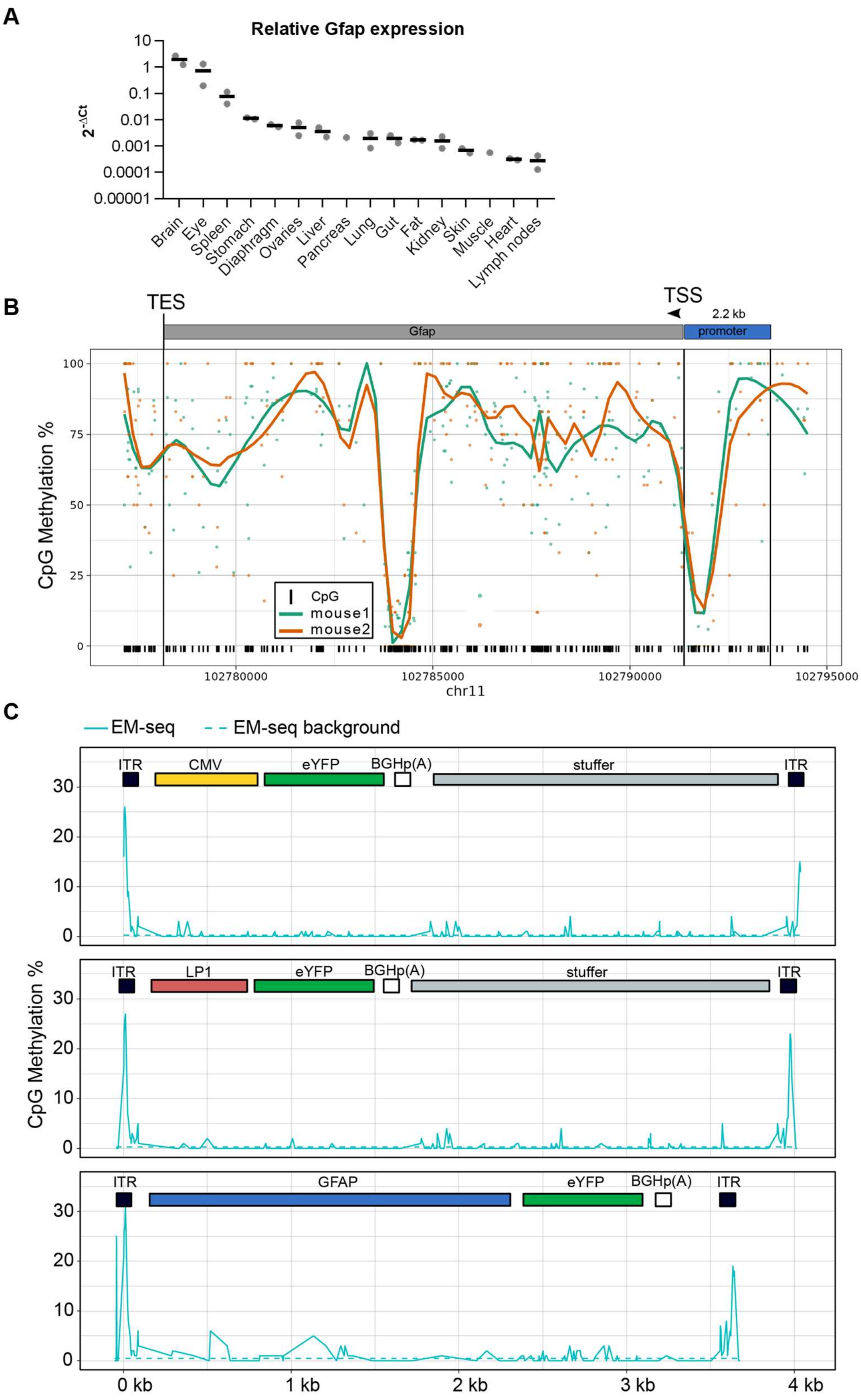

Figure S11. Evaluation of endogenous Gfap expression and methylation status of endogenous or AAV-delivered promoters. (A) Relative expression of the endogenous *Gfap*

normalized to *PolR2A* housekeeper expression. Data from n=2 untreated mice. (B) Liver CpG methylation frequency along the endogenous mouse *Gfap* gene on chromosome 11 of GRCm39 (NC_000077.7:102778162-102791368) as determined from bisulfite sequencing data derived from publicly available data (SRR24770973, SRR24770974) (n=2 untreated mice). The mouse *Gfap* promoter is here defined as the 2.2 kb sequence upstream of the transcription start site (TSS; NC_000077.7:102791368-102793568). Every dot represents the methylation frequency of a single CpG site. The line represents a loss of the individual CpG sites. TES, transcription end site. (C) Methylation frequency along the AAV vector genomes in the liver containing a CMV, LP1, or GFAP promoter as determined 14 days post-delivery through EM-seq. The dotted line represents the mean methylation frequency determined from unmethylated DNA.

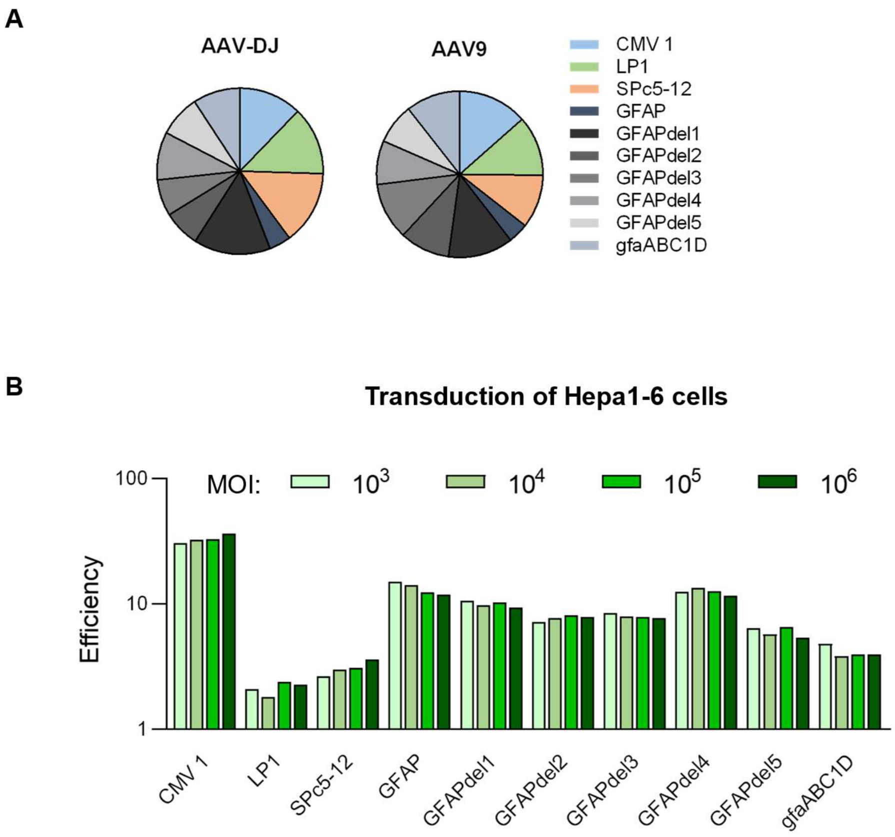

Figure S12. Promoter libraries for the study of GFAP promoter deletion variants. (A) NGS-based characterization of the composition of the AAV-DJ- and AAV9-based promoter mini-libraries. (B) Relative promoter efficiency as determined by barcode sequencing after transduction of Hepa1-6 cells. Hepa1-6 cells were transduced with the AAV-DJ mini-library using different MOIs (10^3, 10^4, 10^5 and 10^6). DNA and RNA were extracted on day 3 post-transduction, followed by barcode readout on DNA and cDNA (RNA) levels. Efficiency scores were calculated as before.

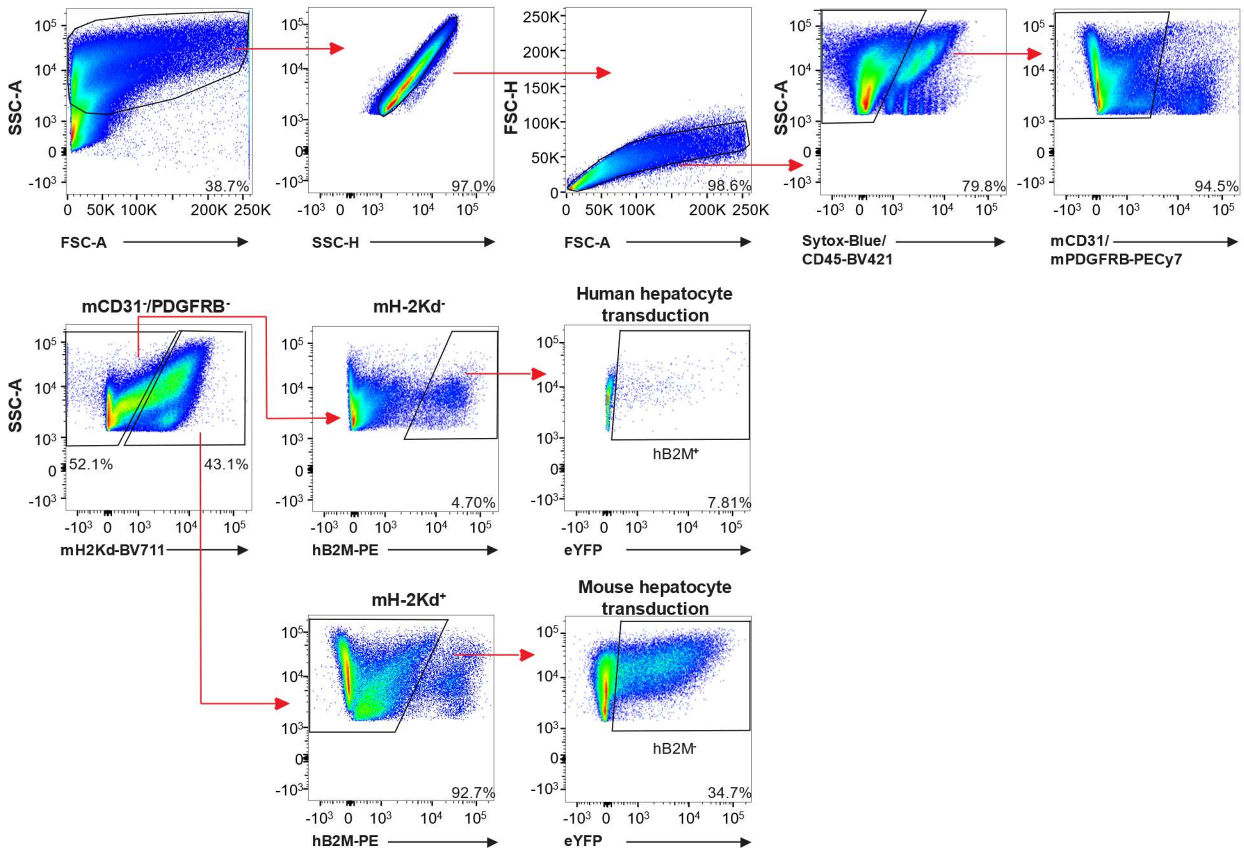

Figure S13. FACS-based isolation of human and murine hepatocytes from FRGN mouse transduced with the AAV9 promoter mini-library. Flow cytometry analysis of human and mouse hepatocytes released from a humanized FRGN mouse two weeks after injection with the AAV9 promoter mini-library. Non-parenchymal cells were excluded using CD45-BV421 and mCD31/mPDGFRB-PECy7 markers. Human hepatocytes were identified and collected from the mH2Kd-BV711-negative, hB2M-PE-positive gate, while mouse hepatocytes were collected from the mH2Kd-BV711-positive, hB2M-PE-negative gate.
